# Adipose-Derived Stem Cell Exosomes Antagonize the Inhibitory Effect of Dihydrotestosterone on Hair Follicle Growth by Activating Wnt/*β*-Catenin Pathway

**DOI:** 10.1155/2023/5548112

**Published:** 2023-09-27

**Authors:** Xin Tang, Cuixiang Cao, Yunxiao Liang, Le Han, Bin Tu, Miao Yu, Miaojian Wan

**Affiliations:** Department of Dermatology, The Third Affiliated Hospital of Sun Yat-Sen University, 600 Tianhe Road, Guangzhou, China

## Abstract

The most prevalent type of alopecia is androgenetic alopecia (AGA), which has a high prevalence but no effective treatment. Elevated dihydrotestosterone (DHT) level in the balding area was usually thought to be critical in the pathophysiology of AGA. The canonical Wnt/*β*-catenin signaling pathway plays a key role in promoting hair follicle development and sustaining the hair follicle cycle. Adipose-derived stem cell exosomes (ADSC-Exos) are widely used in the field of regenerative medicine due to the advantages of being cell free and immune privileged. Still, few studies have reported the therapeutic effect on hair disorders. As a result, we sought to understand how ADSC-Exos affected hair growth and explore the possibility that ADSC-Exos could counteract the hair-growth-inhibiting effects of DHT. This research using human hair follicle organs, in vitro dermal papilla cells, and in vivo animal models showed that ADSC-Exos not only encouraged healthy hair growth but also counteracted the inhibitory effects of DHT on hair growth. Additionally, we discovered that ADSC-Exos increased Ser9 phosphorylated glycogen synthase kinase-3*β* levels and facilitated nuclear translocation of *β*-catenin, which may have been blocked by the specific Wnt/*β*-catenin signaling pathway inhibitor dickkopf-related protein 1. Our findings suggested that ADSC-Exos are essential for hair regeneration, which is anticipated to open up new therapeutic possibilities for clinical alopecia, particularly for the treatment of AGA.

## 1. Introduction

Alopecia is a common disease affecting more than half of the global population, of which androgenetic alopecia (AGA) is the most common type [1]. The prevalence of AGA ranged 30%–70%. The disease has a great negative impact on patients' external image, mental health, life, and work [2]. The treatment of AGA is still a global problem that attracts blooming interest in research. Oral finasteride and topical minoxidil are still the main treatments for AGA. However, long-term use of finasteride has a potential risk of sexual dysfunction, and irritation of minoxidil also affects patient compliance. Therefore, it is encouraged to develop new generative strategies that antagonize androgens, activate Wnt/*β*-catenin pathway, and restore the normal hair follicle cycle with lasting effectiveness and few side effects.

At present, the exact etiology and mechanism of AGA are still unclear, and it is generally believed that genetic factors, locally increased androgen levels and overexpression of androgen receptor (AR) play significant roles in the pathogenesis of AGA [3–5]. Under the action of type II-5*α* reductase, testosterone is transformed into dihydrotestosterone (DHT) with higher activity and binds to AR, leading to the onset of AGA. Hair follicles show periodic development rules, including three stages: anagen, catagen, and telogen. The duration of hair follicles in AGA patients is gradually shortened in anagen, while the duration of telogen is prolonged, and the hair follicles are progressively miniaturized and eventually lead to alopecia [6, 7]. The pathways involved in regulating hair follicle cycle development mainly include Wnt/*β*-catenin, transforming growth factor-*β* (TGF-*β*), bone morphogenetic protein (BMP), sonic hedgehog (SHH) etc. Among them, Wnt/*β*-catenin signaling pathway is essential in hair follicle morphogenesis and primary hair follicle formation [8, 9]. Wnt signaling is a highly conserved pathway that is essential for the development and homeostasis of multicellular organisms [10]. Glycogen synthase kinase-3*β* (GSK-3*β*), which plays a pivotal role in the regulation of self-renewal and function of cell populations [11], can be negatively regulated by Wnt signaling through phosphorylation at residue Ser9, which in turn leads to *β*-catenin stabilization [12]. This signaling pathway is regulated by the stabilization and translocation of the key signal transducer *β*-catenin to the nucleus, where it regulates the expression of renewal and proliferation genes [13]. Current studies suggest that DHT inhibition of the Wnt/*β*-catenin pathway is a key pathological process in the pathogenesis of AGA; therefore, in this study we targeted the Wnt/*β*-catenin signaling pathway to explore new therapeutic options for AGA.

Dermal papilla cells (DPCs) are the “command center” of hair follicle generation and cycle transformation. DPCs locate at the base of hair follicles and is a crucial element in hair follicle development and regeneration. As important markers of DPCs and indicators of hair-inducing ability, alkaline phosphatase (ALP), and versican induce follicle formation when specially highly expressed in DPCs of hair follicles during the anagen phase [14–16]. The expression and secretion of various hair follicle inductive molecules in DPCs have been reported to describe the hair inducibility of DPCs [17]. It expresses AR, receives stimulation from DHT and other factors, and regulates other hair follicle cells in a paracrine manner. Recent studies have shown an interaction between DHT and Wnt/*β*-catenin signaling pathway in the pathogenesis of AGA [18–20]. DHT treatment downregulates the classical Wnt pathway in DPCs and inhibits hair follicle growth. Activating the Wnt signal could restore the DHT-impaired hair follicle inductive ability in DPCs. Therefore, effective activation of the classical Wnt pathway will become a key target in the treatment of AGA.

Exosomes (Exos) are a kind of membranous vesicles with a diameter range 30–150 nm and can be secreted by most cell types [21]. Exos carry various bioactive substances such as lipids, nucleic acids, and proteins, which participate in intercellular communication and regulate cell biological activity [22] While maintaining the same biological activity as the source cells, exosomes can overcome the shortcomings of stem cell therapy and are stable and easy to preserve. More importantly, they can be commercially produced in large quantities, avoiding multiple invasive procedures, and demonstrating promising clinical translation prospects. Adipose-derived stem cells (ADSCs), the most easily obtained mesenchymal stem cells, are essential components of the microenvironment around hair follicles. ADSCs play a vital role in the hair cycle process, stimulating DPCs and promoting the normal hair follicle cycle [23, 24]. Studies have shown that ADSCs can secrete various cytokines to promote hair growth. The treatment of AGA with ADSCs conditioned medium and local injection of stromal vascular fraction rich in ADSCs showed good hair growth effect [25–27]. ADSC-Exos have been widely studied and proved to have strong potential in regenerative medicine, such as nourishing nerve tissue, improving liver fibrosis, promoting bone regeneration, promoting wound healing, and improving angiogenesis etc. [28–31]. ADSC-Exos play a role in treating many physiological processes and diseases by activating the Wnt/*β*-catenin signaling pathway [32–34]. However, few studies have reported on the therapeutic effects of ADSC-Exos in AGA.

In this study, we hypothesized that ADSC-Exos had the same hair growth-promoting activity as ADSCs and could antagonize the inhibitory effect of DHT on hair follicle growth. We also aimed to clarify whether the activation of Wnt/*β*-catenin signaling pathway played an important role and explore the underlying mechanism, to develop new strategies for AGA cell-free therapy.

## 2. Materials and Methods

### 2.1. Cell Culture and Identification

#### 2.1.1. Adipose-Derived Stem Cells

Human ADSCs were isolated from the subcutaneous fat of patients with liposuction, which was consented in accordance with the Ethics Committee at The Third Affiliated Hospital of Sun Yat-Sen University. The chopped fat tissue was digested with 0.2% collagenase type I (Sigma, USA) at 37°C for 30 min. After being centrifuged at 1,000 rpm for 3 min, cell precipitates were resuspended in DMEM with low glucose (1.0 g/L), containing 10% FBS and 1% penicillin–streptomycin, and cultured at 37°C in an incubator of 5% CO_2_. The medium was replaced for 48 hr. Passage 3 ADSCs were used for the following experiments. The identification procedure of ADSCs is detailed in the supplementary materials.

#### 2.1.2. Dermal Papilla Cells

Human DPCs (ScienCell, USA) were purchased from Science Cell Research Laboratories. According to the instructions, DPCs were cultured in mesenchymal stem cell culture medium (ScienCell, USA) containing 5% exosome-free FBS, 1% penicillin–streptomycin, and 1% stem cell growth factor in a 5% CO_2_ incubator at 37°C. The cell passage number 3 was used in this study [35].

### 2.2. Exosomes Isolation and Identification

We used ultracentrifugation in this study to isolate exosomes as previously reported [21, 36]. In brief, when the confluence of ADSCs reached 75%–85%, the medium was replaced with exosome-free serum medium and cultured for 48 hr. Conditioned media was collected to centrifugate at 300 *g* for 10 min, 2,000 *g* for 20 min, and 10,000 *g* for 30 min at 4°C to remove cells and debris; afterward, the supernatant was filtered through a 0.22 *µ*m filter (Merck Millipore, USA). Subsequently, the filtered medium was ultracentrifuged at 100,000 *g*, 70 min twice and exosomes were resuspended with PBS and stored in −80°C for the following experiments.

The particle size and concentration of exosomes were measured by nanoparticle tracking analysis (NTA) with Nanosight NS300 (Malvern, England). Exosome morphology was observed and photographed under the transmission electron microscopy (TEM) (JEM-1200EX, Japan), according to previously reported protocol [37]. The specific positive markers of exosomes TSG101, CD9, and CD81 (Abcam, USA) were detected by the western blotting.

### 2.3. Exosomes Labeling and Uptake Assay

As recommended, exosomes derived from ADSCs were labeled with the green fluorescence dye PKH67 (Sigma-Aldrich, Germany). Then, the PKH67-labeled Exos were obtained after ultracentrifuged again at 100,000 *g* for 70 min to remove excess dye. For the internalization assay, DPCs were incubated with PKH67-labeled exosomes for 24 hr and the results were evaluated by confocal laser scanning microscopy.

### 2.4. Cell Proliferation Assay

The proliferation of DPCs and chondrocytes stimulated by Exos was evaluated by cell counting kit-8 (CCK-8) (Dojindo, Japan). In brief, DPCs were seeded in 96-well plates (5,000 cells/well). After cell adherence, DPCs were divided into ADSC-Exos groups of 0, 5, 10, 20 *μ*g/mL or control, DHT (10^−5^ mol/L), Exos and DHT + Exos groups and treated for 72 hr. Then, 10 *μ*L CCK-8 solution was added to each well for 2 hr at 37°C. The optical density (OD) at 450 nm absorbance values was measured to evaluate the cell proliferation.

### 2.5. Cell Migration Assay

In this study, scratch wound healing assay was used to assess the effect of exosomes on the migration ability of DPCs. DPCs were seeded into 6-well plates (5 × 10^5^ cells/well). When the confluence reached about 90%, uniform scratch wounds were scraped by a 200 *μ*L sterile pipette tip. Subsequently, gently wash away the scratched cells with PBS, and add medium containing different concentrations of ADSC-Exos, DHT (10^−5^ mol/L), Exos, and DHT + Exos or vehicle. Images were observed and taken by microscope at 0, 12, 24, and 48 hr after scratching.

### 2.6. Western Blot

Western blot analysis was performed as a standard protocol. Exosomes, cells, or dorsal skin tissue were lysed by RIPA on ice, and then the protein concentration was measured by a BCA Protein Assay Kit (Thermo Fisher Scientific, USA). The primary antibodies were as follows: anti-phospho-GSK-3*β* (Ser9) (1 : 7,000, Rabbit monoclonal), anti-GSK-3*β* (1 : 7,000, Rabbit monoclonal), anti-*β*-catenin (1 : 7,000, Rabbit monoclonal), anti-Versican (1 : 2,000, Rabbit monoclonal), anti-ALP (1 : 1,000, Rabbit monoclonal), anti-GAPDH (1 : 10,000, Rabbit monoclonal), anti-CD9 (1 : 500, Rabbit monoclonal), anti-CD81 (1 : 500, Rabbit monoclonal), anti-TSG101 (1 : 2,000, Rabbit monoclonal), and anti-calnexin (1 : 3,000, Rabbit monoclonal) (Abcam, USA).

### 2.7. Hair Follicles Separation and Organ Culture

HFs were separated according to a previous study by Langan et al. [38]. In short, after the scalp was cleaned, it was divided into a single hair follicle. The hair follicle was cut off at the dermal–subcutaneous fat junction under a dissecting microscope (Leica Microsystems, Germany). Isolated hair follicles were carefully transferred to supplemented Williams' E medium. The medium was serum-free William's E Medium (Gibco, USA) containing 10 *μ*g/L hydrocortisone (MP Biomedicals, USA), 10 mg/L insulin–transferrin–selenium, 2 mM L-glutamine (Gibco, USA), 2 mM HEPES (Gibco, USA), and 1% antibiotics (streptomycin and penicillin, Gibco, USA). Mediums were replaced every other day. Suspensions were cultured in 5% CO_2_ incubator at 37°C.

After 24 hr of culture, hair follicles at anagen stage VI were selected in this study (Figure S2) [39]. Subsequently, HFs were treated with ADSC-Exos of different concentrations and 10^−5^ mol/L DHT. The vehicle group was considered as the negative control group. HFs morphology was imaged using an inverted microscope; the hair shaft elongation was measured every other day, and the mean of hair shaft growth was reckoned.

### 2.8. Immunofluorescence

Cultured HFs were cut into 3 *μ*m paraffin sections and put on standard microscope slides. Paraffin sections with complete structures were selected and dewaxed. Then, the slides were repaired using EDTA solution (pH 9.0), blocked with goat serum, and incubated with primary antibodies against Ki67, *β*-catenin, GSK-3*β*, and Ser9 phosphorylated glycogen synthase kinase-3*β* (pGSK-3*β*) (Abcam, UK) overnight at 4°C. Secondary antibodies (goat anti-rabbit, Abcam, UK) were added and incubated in the dark for 1 hr. The slides were washed with PBST three times and incubated with 4′, 6-diamidino-2-phenylindole (DAPI; Beyotime Biotechnology, China) in the dark for 10 min. The images were acquired under a fluorescence microscope. ImageJ software (NIH, Bethesda, MD, USA) was used to transform the obtained images into grayscale images, and the average fluorescence intensity was measured.

### 2.9. In Vivo Tracking Experiment

The animal use protocol listed below was reviewed and approved by the Institutional Animal Care and Use Committee, Jenio Biotech Co., Ltd. Exosomes labeled with DiR were used for fluorescence signal tracking on an In Vivo Imaging System (IVIS). Six 7-week-old male C57BL/6 mice were randomized into two groups: PBS (control) group and ADSC-Exos/DiR group. DiR-labeled ADSC-Exos or PBS was subcutaneously injected to the dorsal skin after back depilation. Fluorescence images were taken at 0.5, 24,48, and 72 hr by the VISQUE InVivo Smart-LF Compact Preclinical in (Vie-work, South Korea). The results were analyzed using MISE software, and data are expressed as means ± SD radiant efficiency (photons/s/cm^2^/sr).

### 2.10. Hair Growth In Vivo

Male 7-week C57BL/6 mice were purchased from Charles River (Beijing, China). After acclimatization for 7 days, the dorsal hair of each mouse was clipped to an area of about 2 × 4 cm followed by depilation. The mice were divided into six groups: PBS group (negative control); minoxidil group (3% minoxidil, positive control); Exos group; DHT group (10^−5^ mol/L DHT, AGA group); and DHT + Exos group (AGA treatment group), DHT + minoxidil group, six mice in each group. Each mouse was uniformly injected subcutaneously on the depilated back with a total volume of 300 *μ*L/each (evenly injected at 12 points), and minoxidil was administered topically. The mice were photographed on days 0, 6, 12, 18, and 21, and the changes of skin color and hair growth in the hair loss area on the back of the mice were recorded.

### 2.11. Histological Analysis

For histology, the dorsal skin of mice was harvested and fixed in 4% paraformaldehyde at 4°C, and then the samples were embedded in paraffin blocks. The section thickness of paraffin blocks was 5 *µ*m. Hematoxylin and eosin (HE) staining was for histological analysis of hair growth in vivo. Skin thickness and HFs number were determined using Image-Pro Plus software. All of these were taken from representative regions (at least three fields).

### 2.12. Cell Immunofluorescence Assay

After pretreating the cell crawls with 0.1% gelatin, they were carefully placed into 12-well plates with forceps. Inoculate the DPCs into the 12-well plate and culture overnight to make the cells adhere to the wall. DPCs were successively divided into ADSC-Exos concentration groups of 0, 5, 10, and 20 *μ*g/mL and control group, DHT (10^−5^ mol/L) group, dickkopf-related protein1 (DKK1) group, Exos group, DHT + Exos, and DHT + DKK1 +  Exos group and treated for 24 hr. Cells were fixed with 4% paraformaldehyde, treated with 0.3% TritonX-100 to permeabilize the cell membrane for 20 min. Diluted *β*-catenin (5% BSA, dilution 1 : 300) primary antibody was added and placed at 4°C overnight. The next day, add fluorescent secondary antibody and incubate at room temperature for 1 hr. Finally, add an appropriate amount of DAPI staining solution and incubate for 10 min to avoid light at room temperature. Select the proper channel under a fluorescent microscope to observe and acquire images.

### 2.13. ELISA

DPCs were seeded into 6-well plates at a density of 2.5 × 10^5^ cells per well. After 24 hr, the culture medium was changed and the cells were pretreated with ADSC-Exo or PBS for 12 hr, then followed by stimulation with DHT. The conditional culture medium at 48 hr was collected, centrifugated at 2,000 *g* for 10 min to remove precipitate and then stored in −20°C for further experiment. Different groups of DPCs supernatants were used to culture the HaCaT cells to explore the effect of substances secreted by DPCs on keratinocyte differentiation. The concentrations of IGF-1 and HGF in the supernatant of DPCs were determined by ELISA kits (Boster, Wuhan, China) following the manufacturer's instructions. The OD was measured at 450 nm by a microplate reader.

### 2.14. RNA Isolation and Quantitative Real Time-Polymerase Chain Reaction (PCR)

Total RNA was extracted from different groups of DPCs and HaCaT cells with an RNA extraction kit (HaiGene Biotech, China) and cDNA was synthesized by the reverse transcription (Takara, Dalian, China). Quantitative PCRs were performed as indicated (Takara, Dalian, China). The primer sequences used are shown in Table S2.

### 2.15. Statistics

Data were performed with SPSS 20.0 statistical software and presented as mean ± SD. All experiments were done at least three times. Multiple comparisons among ≥3 groups were performed using one-way ANOVA. The differences were considered statistically significant when *P* < 0.05.

## 3. Results

### 3.1. Characterization of ADSCs and ADSC-Exos

**Figure 1:**
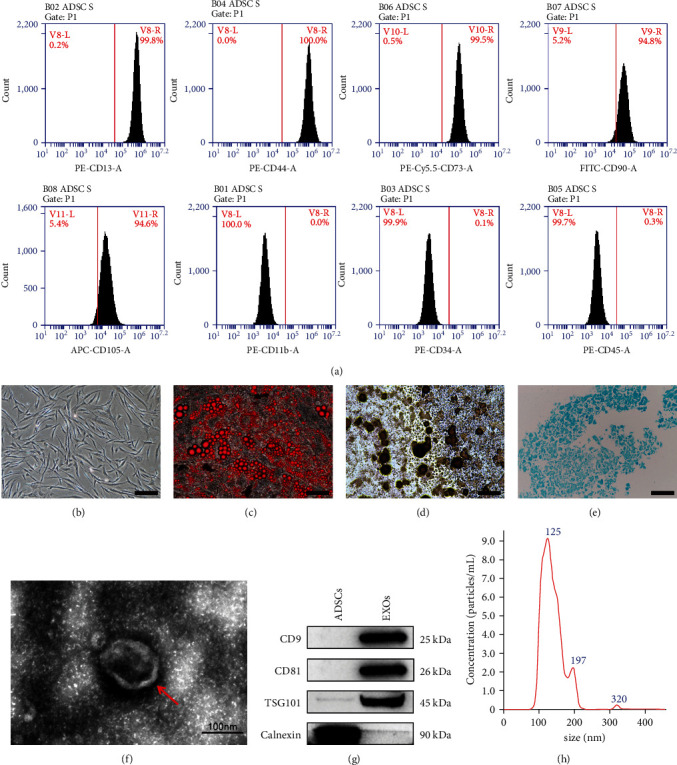
Identification of ADSCs and ADSC-Exos. (a) Detection of surface markers of ADSCs by flow cytometry. (b) Cell morphology of human adipose stem cells, scale bar: 200 *μ*m. (c) Lipogenic differentiation (Oil Red O staining), scale bar: 100 *μ*m. (d) Osteogenic differentiation (alizarin red staining), scale bar: 100 *μ*m. (e) Chondrogenic differentiation (alcian blue staining), scale bar: 100 *μ*m. (f) Morphology of ADSC-Exos observed by transmission electron microscopy, scale bar: 100 nm. (g) Detection of exosome surface markers by western blot. (h) ADSC-Exos particle size distribution by NTA.

The extracted cell types were identified according to the three criteria defining ADSCs proposed by the International Federation for Adipose Therapeutics and Science and the International Society for Cellular Therapy: (1) plastic adherence, (2) cell surface markers, and (3) differentiation potential [40, 41]. The positive markers of CD13, CD44, CD73, CD90, and CD105 were highly expressed, while the negative markers of CD11b, CD34, and CD45 were almost not expressed (Figure 1(a)). In terms of cell morphology and plastic adherence ability, ADSCs were spindle-shaped and showed adnexal growth, which was consistent with the morphology of mesenchymal stem cells (Figure 1(b)). The cells we isolated differentiated into adipocytes, osteoblasts, and chondroblasts (Figure 1(c)–1(e)). According to the previous studies by Zhao et al. [36], TEM, western blot, and NTA were used to characterize the purified nanoparticles from ADSCs. ADSC-Exos under TEM were completed in shape, showing a spherical or round cup shape (Figure 1(f)). ADSC-Exos had higher expression of CD9, C81, and TSG101 proteins compared with ADSCs cell lysates, but did not express the cytoplasmic endoplasmic reticulum protein Calnexin (Figure 1(g)). The main particle size peak of NTA exosomes was around 120 nm, which was in the range of 30–150 nm (Figure 1(h)).

### 3.2. ADSC-Exos Increase the Proliferation, Migration, and Hair Inducibility of DPCs

**Figure 2:**
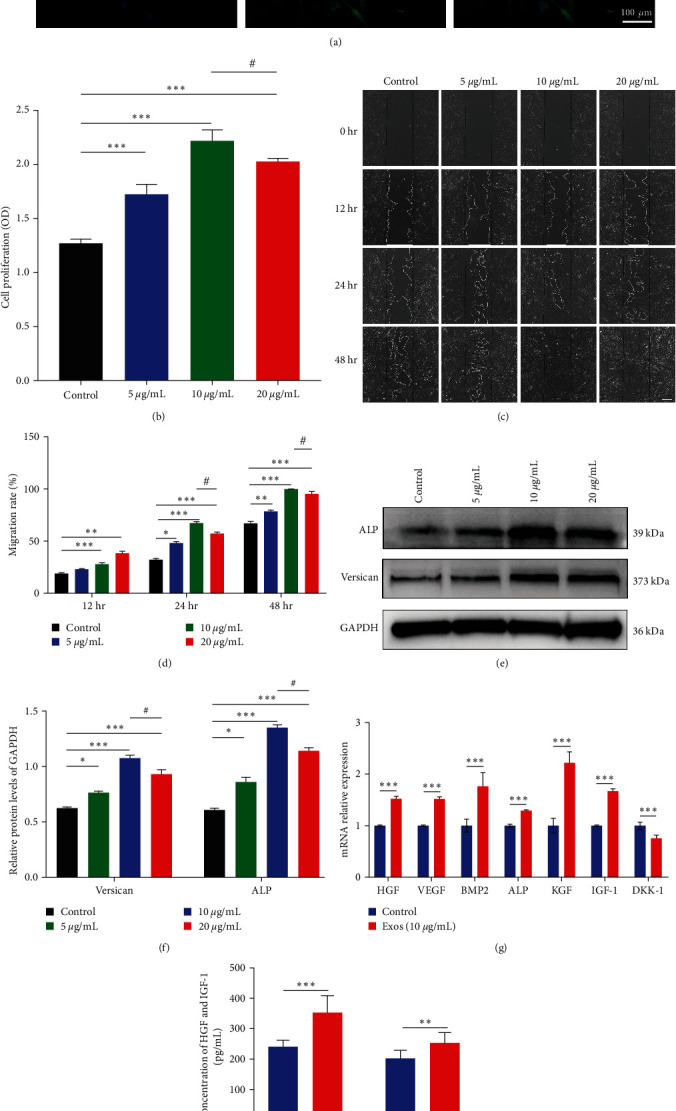
ADSC-Exos promotes the proliferation and migration of DPCs and enhances hair inducibility. (a) Uptake of PKH67 fluorescently labeled ADSC-Exos by dermal papilla cells. Blue fluorescence: DAPI-stained nuclei; green fluorescence: PKH67-labeled ADSC-Exos, scale bar: 100 *μ*m. (b) CCK-8 absorbance values of DPCs treated with different concentrations of ADSC-Exos at 24, 48, and 72 hr. (c) Scratch experimental plots of ADSC-Exos treated DPCs, scale bar: 100 *μ*m. (d) Quantification of scratch area in Figure 4(b). (e) Graph of versican, ALP protein bands. (f) Relative quantitative graph of protein bands with grayscale values. (g) qPCR result of VEGF, HGF, BMP2, ALP, KGF, DKK-1, and IGF-1. The expression level of target genes was standardized to that of GAPDH. (h) Expression of HGF and IGF-1 detected by ELISA in the culture supernatant of DPCs; *N* = 3,  ^*∗*^*P* < 0.05,  ^*∗∗*^*P* < 0.01,  ^*∗∗∗*^*P* < 0.001, and #*P* < 0.05, 10 *μ*g/mL compared with 20 *μ*g/mL.

Confocal immunofluorescence imaging showed that PKH67 labeled ADSC-Exos could aggregate in DPCs (Figure 2(a)). These results indicate that ADSC-Exos can be successfully ingested and internalized by DPCs.

To investigate the effects of different concentrations of ADSC-Exos on the proliferation activity of DPCs, DPCs were treated with 0,5, 10, and 20 *μ*g/mL ADSC-Exos. Compared with the control group, ADSC-Exos group showed higher proliferation, and the promoting effect increased with the increase of concentration within a certain range, showing concentration dependence. However, when the concentration reached 20 *μ*g/mL, the promoting effect was not as significant as 10 *μ*g/mL, indicating that 10 *μ*g/mL is the most suitable concentration (Figure 2(b)). Similar results were obtained for the effect of ADSC-Exos on the migration capacity (Figures 2(c) and 2(d)) and hair-inducing ability of DPCs (Figure 2(e)–2(h)). ALP and Versican, as important markers of hair inducibility, were significantly elevated after treatment of ADSC-Exos (Figures 2(e) and 2(f)). Similarly, qPCR and ELISA results revealed that ADSC-Exos increased expression and secretion of hair follicle inductive molecules in DPCs (Figures 2(g) and 2(h)). Moreover, cell-free medium from exosomes-pretreated DPCs possessed stronger ability to induce differentiation of keratinocytes (Figure S4). Thus, ADSC-Exos promote the proliferation, migration, and hair inducibility of DPCs and 10 *μ*g/mL was an appropriate concentration.

### 3.3. ADSC-Exos Promote Hair Follicle Growth and Prolong Anagen in Organ Culture

**Figure 3:**
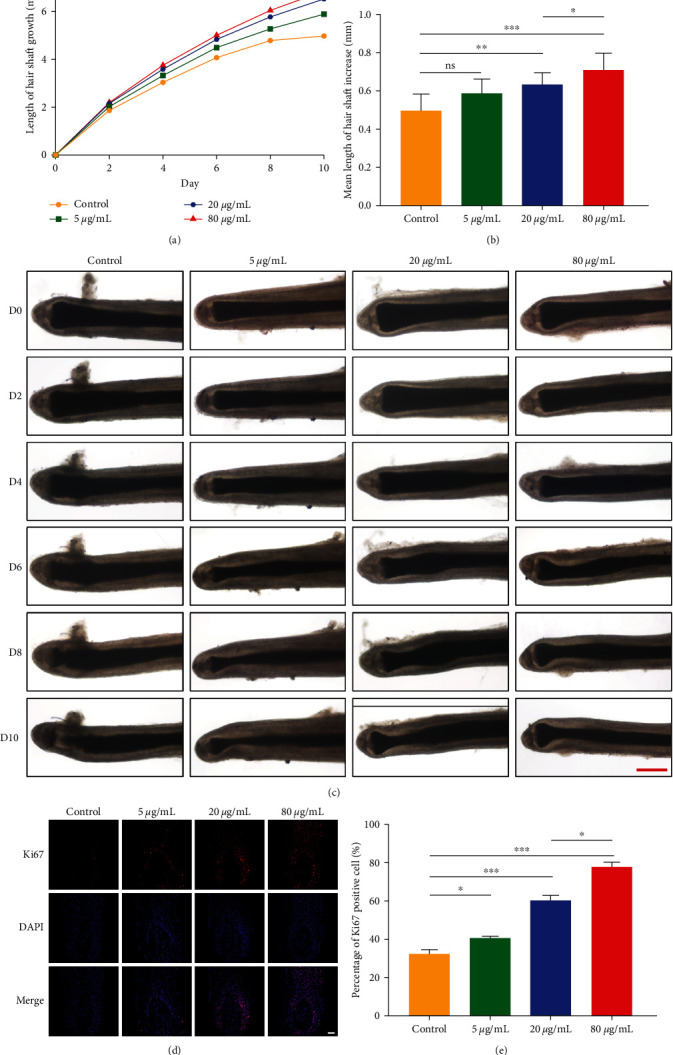
ADSC-Exos promotes the elongation of human hair follicles and prolongs anagen. (a) Hair follicle growth graph, *n* = 6. (b) Mean length of hair shaft increase (mm). *n* = 6. (c) Morphology of hair follicle growth at 0, 2, 4, 6, 8, and 10 days after ADSC-Exos treatment, scale bar: 500 *μ*m. (d) Ki67 expression of hair follicles in each group, scale bar: 100 *μ*m. (e) The proportion of Ki67+ hair follicle cells in each group, ns: *P* > 0.05;  ^*∗*^*P* < 0.05,  ^*∗∗*^*P* < 0.01, and  ^*∗∗∗*^*P* < 0.001.

Human scalp hair follicle culture is a good organ model for hair preclinical studies. Thus, it has been widely used to assess the hair growth promoting effect of various factors. In order to investigate the effects of ADSC-Exos on the growth of human hair follicles, human hair follicles were cultured with 5, 20, and 80 *μ*g/mL ADSC-Exos. The results showed that the hair follicle growth was good and the hair shaft was prolonged. Compared with the control group, the hair shaft elongation and average growth rate in all exosome groups increased in a concentration-dependent range, among which 80 *μ*g/mL ADSC-Exos had the most apparent promoting effect (Figures 3(a) and 3(b), Table S1).

The morphological changes in the hair papilla and hair matrix area can be used to distinguish the anagen and regression phases of the hair follicles. In the early stage of catagen, hair follicles undergo a series of morphological changes, such as thinning of the hair matrix, more elliptic dermal papilla and decreased melanin [39]. The control group entered the regression phase from the 6th day, the hair matrix became fine, the melanin decreased, and the dermal papilla was separated from the hair fiber and hair matrix on the 8th day. While the 20 and 80 *μ*g/mL ADSC-Exos group hair follicles began to gradually develop catagen-like morphological changes until the 10th day (Figure 3(c), Figure S3), implying that ADSC-Exos treatment could stimulate hair growth of scalp hair follicles and delayed hair follicle degeneration.

Ki67 is a proliferative nuclear antigen reflecting cell proliferation [42]. Hair matrix keratinocytes are a group of rapidly proliferating cells. In anagen, Ki67+ hair matrix keratinocytes proliferate and differentiate into various hair follicle cells to achieve hair stem extension, while in the resting phase, Ki67 is almost not expressed [39]. Therefore, the positive proportion of Ki67 in the hair matrix is an important indicator in evaluating the proliferation of hair follicles. The expression of Ki67 in hair follicles was significantly increased after ADSC-Exos treatment. The proportion of Ki67+ cells was 32.1% in the control group, and the proportion of Ki67+ cells in 5 *μ*g/mL (40.5%), 20 *μ*g/mL (60.3%), and 80 *μ*g/mL (77.8%) ADSC-Exos groups was significantly increased, among which 80 *μ*g/mL groups had a most significant promoting effect (Figures 3(d) and 3(e)).

These results indicate that ADSC-Exos can significantly promote the hair shaft elongation, prolong hair follicle anagen, and expedite hair follicle growth.

### 3.4. ADSC-Exos Antagonizes the Inhibitory Effect of DHT on DPCs and Hair Follicles

**Figure 4:**
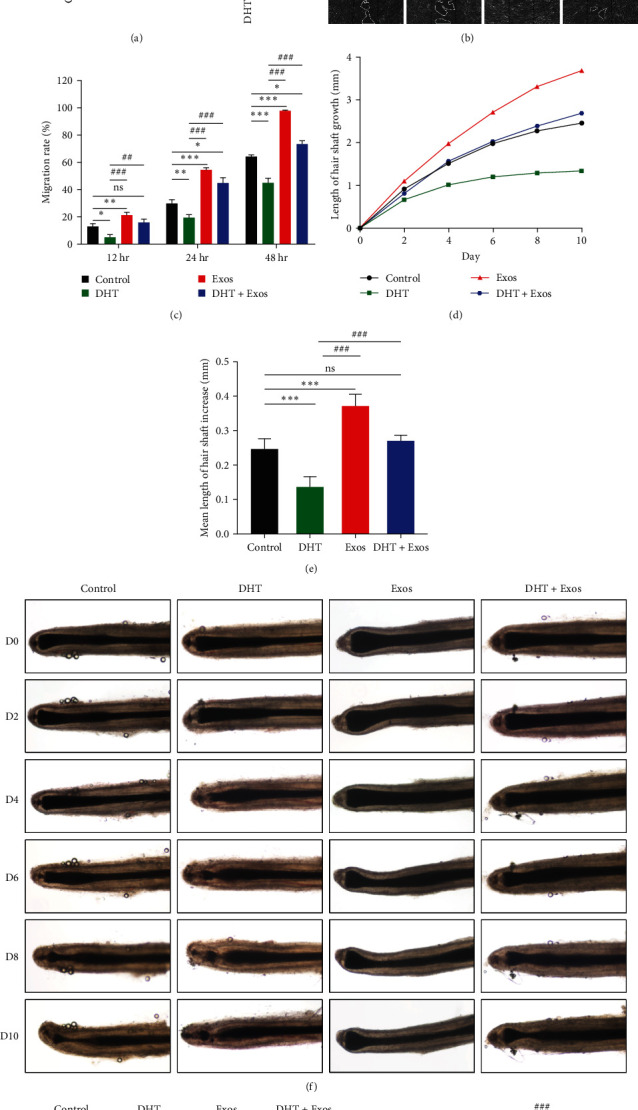
ADSC-Exos antagonizes the inhibitory effect of DHT on hair follicle growth. (a) CCK-8 absorbance values of ADSC-Exos and/or DHT treated DPCs after 72 hr. (b) Scratch experiment of ADSC-Exos and/or DHT treated DPCs for 0, 12, 24, and 48 hr, scale bar: 100 *μ*m. (c) Quantitative analysis of scratch area in (d) Hair follicle growth graph, *n* = 6. (e) Mean length of hair shaft increase (mm), *n* = 6. (f) Morphology of hair follicle growth at 0, 2, 4, 6, 8, and 10 days after ADSC-Exos and/or DHT treatment, scale bar: 500 *μ*m. (g) Ki67 expression of hair follicles in each group, scale bar: 100 *μ*m. (h) The proportion of Ki67+ hair matrix keratinocytes in each group.  ^*∗*/#^*P* < 0.05,  ^*∗∗*/##^*P* < 0.01,  ^*∗∗∗*/###^*P* < 0.001. Comparison with  ^*∗*^control group and ^#^DHT group.

Given the central role of DHT in AGA in vivo and the inhibitory effect in vitro [43, 44], we further investigated whether ADSC-Exos could antagonize the inhibitory effect of DHT and thus treat AGA. The DPCs were divided into the control group, DHT group, Exos group, and DHT + Exos group. CCK-8 experiment showed that after the addition of ADSC-Exos, the proliferation activity of DPCs in the DHT + Exos group was significantly higher than that in the DHT group (Figure 4(a)). Similar results were obtained in the scratching experiments. In the ADSC-Exos treatment group, cells on both sides of the scratch were completely fuzed at 48 hr. The migration rate was 73.2% in the DHT + Exos group and 64.5% in the control group, and only 45% in the DHT group. The migration activity was significantly improved in the DHT + Exos group compared to the DHT group (Figures 4(b) and 4(c)).

We continue to explore the therapeutic effect of ASDC-Exos on AGA in hair follicles. After the addition of ADSC-Exos, the hair follicle growth of the DHT + Exos group was significantly improved compared with the DHT group (Figure 4(d)–4(f), Figure S3, and Table S1). In the DHT group, the hair matrix area began to thin on the 4th day, with degenerative changes and gradual separation of the dermal papilla from the hair fibers and hair matrix, while the control group and the DHT + Exos group entered the degeneration stage from the 6th day. Similar results were obtained with Ki67 immunofluorescence staining of hair follicles to detect the proliferation of hair matrix keratinocytes. Compared with the control group, the proportion of Ki67+ of matrix keratinocytes was increased in the Exos group (69.4%) but significantly decreased in the DHT group (12.5%). There was no significant difference between the DHT + Exos group (39.7%) and the control group (33.1%). The proliferation activity of hair matrix keratinocytes in the DHT + Exos group was significantly higher than that in the DHT group (Figures 4(g) and 4(h)).

These results indicate that ADSC-Exos antagonizes the inhibitory effect of DHT on hair follicle growth and restores normal hair follicle growth.

### 3.5. ADSC-Exos Increase Skin Thickness and the Number of Hair Follicles, Accelerating the Telogen-to-Anagen Transition

We detected the retention of ADSC-Exos in C57BL/6 mice using DiR-labeled ADSC-Exos in vivo fluorescence imaging and determined the interval of administration is 48 hr. (Figure S1)

To determine whether ADSC-Exos can induce hair regeneration and antagonize the inhibitory effect of DHT in vivo, we used 7-week-old male C57BL/6 mice to simulate the AGA model by subcutaneous injection of DHT. 7-week-old C57BL/6 mice entered the synchronous telogen after depilation and the skin was pink during the rest period, while the skin gradually darkened with a new anagen initiation [45]. The mice were treated with 3% Minoxidil (positive control) [46], DHT, Exos, DHT + Exos, DHT + Minoxidil, and control (vehicle, negative control).

**Figure 5:**
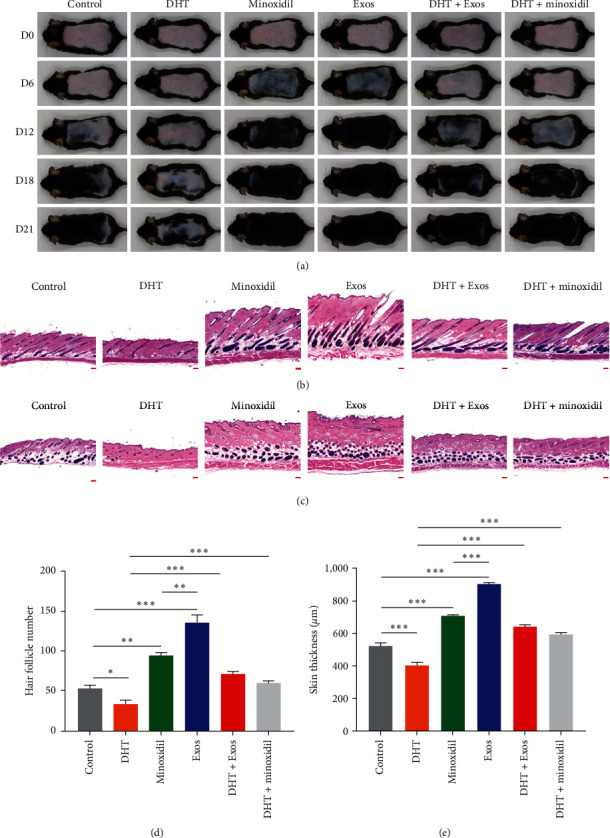
ADSC-Exos increased skin thickness and the number of hair follicles, and accelerated telogen-to-anagen transition. (a) The 7-week-old C57/BL6 mice were randomly divided into control, DHT, Minoxidil, Exos, DHT + Exos and DHT + Minoxidil groups for treatment after dorsal depilation. The observation period was 21 days, and pictures were taken on days 0, 6, 12, 18 and 21, 6 in each group. (b) HE staining of dorsal skin of mice in each group. Longitudinal section of skin, scale bar: 200 *μ*m. (c) HE staining of dorsal skin of mice in each group. Transverse section of skin, scale bar: 200 *μ*m. (d) Quantitative comparison of skin thickness in each group of mice. (e) Comparison of the number of hair follicles in each group of mice ( ^*∗*^*P* < 0.05,  ^*∗∗*^*P* < 0.01, and  ^*∗∗∗*^*P* < 0.001).

As shown in Figure 5(a), the skin of the Exos group became diffuse gray on the 6th day, and a considerable part of the skin of the minoxidil group became gray, indicating that it gradually entered anagen, while the skin of mice in other groups remained pink. On Day 21, hair was completely regenerated in the Exos group, the hair growth of mice in the Exos and minoxidil groups was significantly better than in the control group, and hair regrowth in the DHT group was significantly inhibited, but the hair in the DHT + Exos and DHT + Minoxidil group was improved compared with the DHT group. Besides, the skin thickness and hair follicle number in all groups were measured (Figure 5(b)–5(e), Figure S5). Compared with the control group, the skin thickness and the number of hair follicles were clearly increased in the Exos and minoxidil groups, and the increase was more pronounced in the Exos group than in the minoxidil group.

These results suggest that ADSC-Exos can effectively promote the transition from telogen to anagen and induce hair regeneration in mice, and the effect is superior to that of minoxidil. After DHT treatment, the telogen of mice was prolonged and the entry into the anagen was delayed, which was similar to that of AGA. However, after ADSC-Exos treatment, the inhibitory effect of DHT on hair follicle growth could be partially reversed and normal hair follicle growth was restored.

### 3.6. ADSC-Exos Activates the Wnt/*β*-Catenin Pathway by Promoting pGSK-3*β* (Ser9) and *β*-Catenin Expression and *β*-Catenin Nuclear Translocation

**Figure 6:**
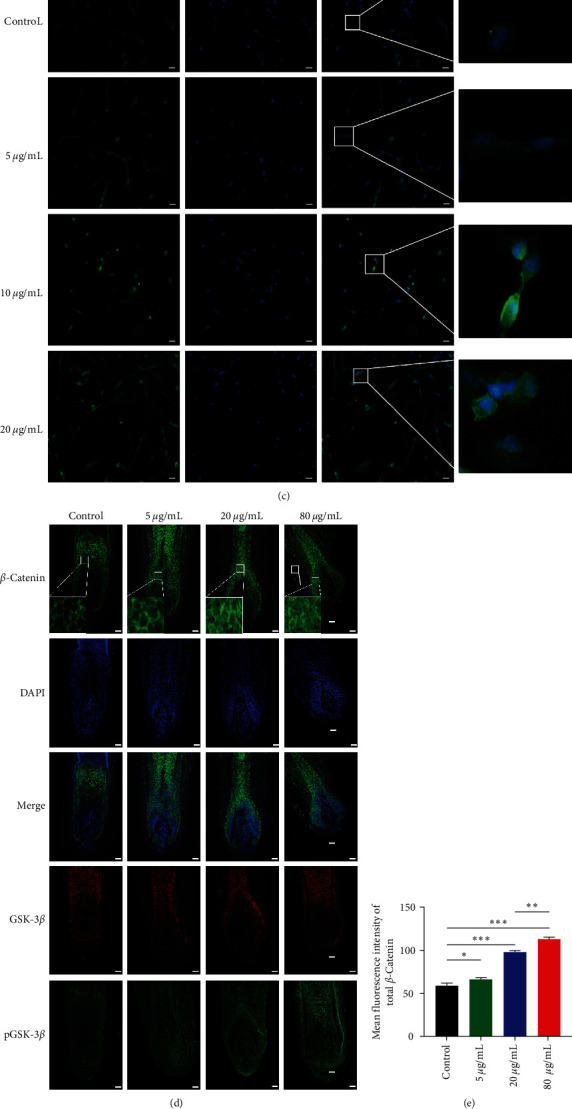
ADSC-Exos activates the Wnt/*β*-catenin pathway by promoting pGSK-3*β* (Ser9) and *β*-catenin expression and *β*-catenin nuclear translocation. (a) ADSC-Exos upregulates the expression of pGSK-3*β* and *β*-catenin. (b) Relative quantification of protein bands in grayscale values. (c) Immunofluorescence staining of *β*-catenin after treatment of DPCs with 0, 5, 10, 20 *μ*g/mL of ADSC-Exos, scale bar: 50 *μ*m. (d) Expression levels of GSK-3*β*, pGSK-3*β*, and *β*-catenin and nuclear transfer of *β*-catenin in hair follicles of each group by immunofluorescence, scale bar: 100 *μ*m. (e–g) Mean fluorescence intensity of total *β*-catenin, nuclear *β*-catenin, and pGSK-3*β*/ GSK-3*β* in hair matrix region. (ns: *P* > 0.05,  ^*∗*^*P* < 0.05,  ^*∗∗*^*P* < 0.01, and  ^*∗∗∗*^*P* < 0.001).

The Wnt/*β*-catenin signaling pathway is a key pathway in hair follicle formation, development, and regeneration. To clarify the potential mechanism of ADSC-Exos therapeutic effect, we examined the changes of the Wnt/*β*-catenin signaling pathway in DPCs and hair follicles after ADSC-Exos treatment. Western blot results showed that compared with the control group, the expression of pGSK-3*β* and *β*-catenin increased significantly with ADSC-Exos treatment, 10 *μ*g/mL is a more appropriate concentration (Figures 6(a) and 6(b)). Immunofluorescence results suggested that *β*-catenin was mainly expressed in the cytoplasm of DPCs in the control group, while the total *β*-catenin expression was higher in each ADSC-Exos concentration group and was expressed more concentrated in the nucleus (Figure 6(c)). We also obtained consistent results in immunofluorescence experiments on hair follicles (Figure 6(d)–6(g)). The above results suggest that ADSC-Exos activates the Wnt/*β*-catenin signaling pathway.

### 3.7. ADSC-Exos Antagonizes the Inhibitory Effect of DHT by Activating the Wnt/*β*-Catenin Pathway

**Figure 7:**
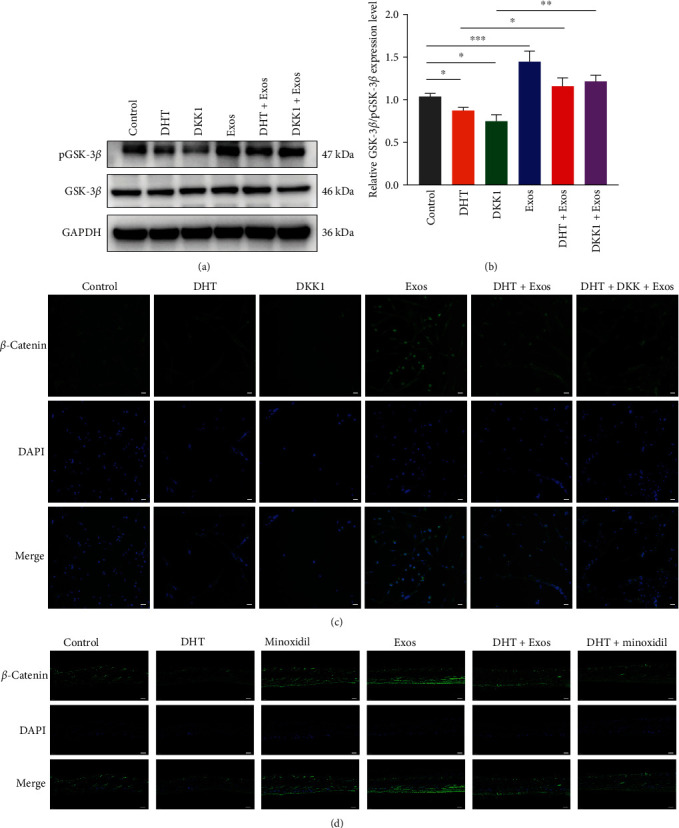
ADSC-Exos antagonizes the inhibitory effect of DHT by activating the Wnt/*β*-catenin pathway. (a) ADSC-Exos partially reverses the inhibitory effect of DHT on pGSK-3*β*. (b) Relative quantification of protein bands in grayscale values. (c) Immunofluorescence staining of *β*-catenin after treatment of DPCs with DHT, ADSC-Exos, DKK1, DHT + Exos, DHT + DKK1 + Exos, and control (vehicle), respectively, scale bar: 50 *μ*m. (d) Immunofluorescence staining of *β*-catenin of C57/BL6 mice skin, scale bar: 200 *μ*m. ( ^*∗*^*P* < 0.05,  ^*∗∗*^*P* < 0.01, and  ^*∗∗∗*^*P* < 0.001).

Previous studies found that DHT in AGA patients downregulated the Wnt/*β*-catenin pathway in hair follicles in the alopecia area [43]. To further confirm the effect of ADSC-Exos on Wnt/*β*-catenin signaling pathway, DKK1, a commonly used inhibitor of this pathway, was used. DKK1 can enhance the *β*-catenin degradation complex to activate GSK-3*β*, reduce the level of pGSK-3*β*, reduce the stability of *β*-catenin, promote its degradation and inhibit its nuclear transfer. Compared with the control group, the phosphorylation levels of GSK-3*β* (pGSK-3*β*/GSK-3*β*) in the DHT and DKK1 groups were significantly decreased, which was increased considerably in the Exos group, and there was no significant difference between the DHT + Exos and DKK1 + Exos groups and the control group (Figures 7(a) and 7(b)). In addition, the effect of ADSC-Exos on DHT can be partially inhibited by DKK1(Figure S6). Consistent results were also obtained in cellular immunofluorescence. In the control group, *β*-catenin was mainly expressed in the cytoplasm of DPCs, and a small amount of *β*-catenin was expressed in the nucleus *β*-catenin expression was significantly reduced in the DHT and DKK1 groups, and was almost absent in the nucleus, while *β*-catenin was mainly expressed in the nucleus in the Exos group. Compared with DHT group, the expression of *β*-catenin was increased in DHT + Exos groups, which was inhibited by DKK1 to a certain extent (Figure 7(c)). In addition, immunofluorescence staining showed that DHT decreased the expression of *β*-catenin in mice skin, which could be partly restored by ADSC-Exos treatment (Figure 7(d)). Therefore, these results revealed that ADSC-Exos antagonize the inhibitory effect of DHT by activating the Wnt/*β*-catenin pathway.

## 4. Discussion

AGA as a common disease, has a severe impact on the mental health of patients. Current treatments are difficult to meet the needs of patients due to various limitations. In this study, we found that ADSCs-Exos antagonized the inhibitory effect of DHT on hair follicle growth by activating Wnt/*β*-catenin pathway and promoting hair regrowth (Figure S7).

Studies have shown that ADSCs can secrete various growth factors, and the application of adipose-derived stem cells, conditioned medium, and stromal vascular fraction rich in ADSCs in the treatment of alopecia has been reported continuously [23, 25, 47]. However, previous studies on the promotion of hair growth by adipose stem cells are mainly cells and conditioned media without the relevant mechanism of action, and there are limitations of stem cells and culture media therapy, such as immune rejection, short survival time, and low overall content. Therefore, ADSCs-Exos used in this study can overcome the above limitations. ADSCs were isolated from adipose tissue by classical enzyme digestion method and the ultra-centrifugation method was used to obtain exosomes and identified them by morphology, particle size, and surface markers, which was consistent with previously reported results [21, 41]. This indicated that we successfully isolated and cultured ADSCs and obtained pure ADSCs-Exos. Its advantages of mass production and less ethical restrictions laid a reliable foundation for subsequent clinical transformation therapy and tissue engineering.

DPCs are the regulatory center of hair growth, and the proliferation of DPCs is necessary for hair follicle morphogenesis and growth. Extracellular vesicles from mouse bone marrow mesenchymal stem cells could promote the proliferation and migration of DPCs, and their promoting effect was dose-dependent in extracellular vesicles [48]. In our study, ADSC-Exos promoted hair growth in a concentration-dependent manner within a certain concentration range, which was consistent with the previous results. When the concentration is too high, its promoting effect is weakened. Likewise, we cultured human hair follicles with different concentrations of ADSC-Exos and found that ADSC-Exos promotes the proliferation of hair matrix keratinocytes and prolongs the hair follicle anagen phase, which leads to the lengthening of the hair shaft. Within a certain concentration range, the higher the concentration of ADSC-Exos, the more obvious the promotion effect. However, the appropriate concentration of ADSC-Exos for promoting human hair follicle growth has not been explored yet. Other cell-derived exosomes also have suitable concentrations for hair follicle growth promotion [49]. In our study, 80 *μ*g/mL of ADSC-Exos was the most suitable promotion concentration. We speculate that there are also a range of appropriate action concentrations for exosomes, which may be related to the limited number of corresponding receptors in the effector cells, and when saturation is reached, increasing the concentration does not lead to further positive effects. In addition, differences in suitable concentrations may be related to different cell types or quantification methods. Different adaptation concentrations also exist at the cellular, organ, and in vivo levels.

At present, only a few studies on hair growth promotion by ADSC-Exos have been reported [43−45]. They have only been explored at the single cellular level or at the animal level under normal hair growth conditions. However, studies on specific hair loss diseases are still scarce. DHT is a critical factor that inhibits hair growth and leads to AGA, and many studies have used DHT to study the related mechanism and treatment of AGA [18, 44], the most common hair loss type. This study established an AGA model with 10^−5^ mol/L DHT [18, 50] at multiple levels in cells, organs, and in vivo to explore whether ADSC-Exos could antagonize the inhibitory effect of DHT on hair growth and achieve the treatment of AGA. We treated DPCs and hair follicles in vitro with DHT, which inhibited the proliferation and migration of DPCs, inhibited the growth of hair follicles and the proliferation of matrix keratinocytes, and shortened the growth period. Treatment with ADSC-Exos could effectively reverse the inhibition effect. We also obtained similar results in C57BL/6 mice. ADSC-Exos had better hair growth promotion results than minoxidil, which were considered a positive control. After DHT treatment, hair growth was inhibited, the growth period was shortened, and the resting period was prolonged, which can better reflect the pathological state of AGA. After ADSC-Exos treatment, skin thickness and hair follicle number were increased in C57BL/6 mice, and the hair cycle was promoted to transition from telogen to anagen.

Wnt/*β*-catenin signaling pathway plays a crucial role in hair follicle morphogenesis and regeneration [10]. *β*-Catenin activity in the dermal papilla regulates hair morphogenesis and regeneration, and the Wnt/*β*-catenin signaling pathway is essential for coordinating mesenchymal–epithelial cell signal interaction of the hair follicles [51]. Activation of Wnt/*β*-catenin pathway can prevent *β*-catenin degradation and transport *β*-catenin to the nucleus, promote the expression of growth-related genes, and play an important role in the initiation of anagen [7]. In addition, DHT inhibition of Wnt/*β*-catenin pathway is a key pathological process in the pathogenesis of AGA. It has been reported that umbilical cord and adipose mesenchymal stem cell exosomes promote wound healing and angiogenesis by activating the Wnt/*β*-catenin signaling pathway [52, 53]. In addition, dermal exosomes promote hair regeneration by regulating *β*-catenin signaling [54, 55]. After ADSC-Exos treatment, we found that the level of a key molecule of Wnt/*β*-catenin pathway, pGSK-3*β* increased, and *β*-catenin also showed higher total level and nuclear translocation level, suggesting that the activation of Wnt/*β*-catenin signaling pathway may be related. DHT downregulates the protein expression of this pathway, which is similar to DKK1, a specific inhibitor of Wnt/*β*-catenin signaling pathway. The addition of ADSC-Exos can partially offset the inhibitory effect of DHT and DKK1 on Wnt/*β*-catenin signaling pathway. It was concluded that ADSC-Exos targeting the main pathological processes and key signaling pathways of AGA is expected to be a better therapeutic strategy. ADSC-Exos contain a large number of functional substances such as RNAs, proteins, and lipids, which mediate the wound repair accelerating, hair growth promotion, and immunomodulatory effects in the various reports [28, 56, 57]. Exosomes-mediated transfer of active ingredients to target cells has been shown to play a crucial role in these effects. However, which component in ADSC-Exos may play a role in activating Wnt/*β*-catenin signaling pathway still remains unspecified and needs further exploration. Even though numerous previous studies have shown that a variety of effective substances (e.g., miR-424-5p, miR-1260b, miR-127-3p, miR-218-5p, miR-22-5p, miR-181a-5p, and miR-148b-3p [54, 58]; protein Hic-5, UBR2, and STAT1 [59–65]) in exosomes from the different sources can regulate the Wnt/*β*-catenin signaling pathway, single miRNA or protein from exosomes might not completely recapitulate the regulatory effects. Therefore, in the present study, instead of exosomal miRNAs or proteins, we used exosomes as a whole to investigate the therapeutic effects on AGA.

There are also some limitations to our study. Aside from the Wnt/*β*-catenin signaling pathway, ADSC-Exos may promote hair growth via a variety of other pathways such as angiogenesis, immunomodulation, the TNF-*α* pathway, and so on. This study, however, provides a strong theoretical support as well as an important preclinical foundation for ADSC-Exos treatment of AGA. It is expected to provide a type of AGA cell-free therapy that is suitable for both men and women and has fewer side effects. It will be the basis for a more in-depth mechanism study of ADSC-Exos in the future.

## 5. Conclusions

ADSC-Exos can enhance hair follicle induction ability, extend the hair follicle growth period, and promote hair regeneration. ADSC-Exos can promote hair growth by activating Wnt/*β*-catenin pathway, antagonize the inhibitory effect of DHT on hair follicle growth, and restore normal hair follicle growth, potentially opening up a new treatment avenue for AGA.

## Data Availability

The data that support the findings of this study are available from the corresponding author upon reasonable request.

## Supplementary Materials

Supplementary Materials Figure S1: retention of ADSC-Exos in C57BL/6 mice. Figure S2: qualitative morphological criteria to distinguish between anagen VI and catagen. In hair follicle organ culture anagen VI hair follicles show a hair matrix with a larger volume, a dermal papilla which is more onion-shaped and a melanin content which is maximal whereas catagen hair follicles have a thinner hair matrix, a dermal papilla which is often more oval and reduced melanin content. The melanin content of anagen hair follicles is higher than in catagen follicles. The percentage of Ki67+ cells of hair matrix keratinocytes in anagen VI hair follicles is significantly augmented. Figure S3: (a) the whole picture of hair shafts of Figures 3(c) and 4(f). Figure S4: HaCat cells were cultured with conditioned medium from DPCs treated with ADSC-Exos. Differentiation markers KRT40, MSX2, KRT5, and KRT15 were detected by qPCR; *N* = 3,  ^*∗*^*P* < 0.05,  ^*∗∗*^*P* < 0.01, and  ^*∗∗∗*^*P* < 0.001. Figure S5: HE images with a full review of the injection sites of Figures 5(b) and 5(c). Figure S6: ADSC-Exos partially reverses the inhibitory effect of DHT on pGSK-3*β*. Figure S7: graphical abstract: ADSC-Exos were isolated from human adipose-derived stem cells by ultracentrifugation. ADSC-Exos promote the proliferation, migration and hair inducibility of DPCs, enhance the elongation of human hair follicles and accelerated telogen-to-anagen transition of C57/BL6 mice. ADSC-Exos counteracted the inhibitory effects of DHT on hair growth. Additionally, ADSC-Exos increased Ser9 pGSK-3*β* levels and facilitated nuclear translocation of *β*-catenin, which indicated that ADSC-Exos antagonize the inhibitory effect of DHT on hair follicle growth by activating Wnt/*β*-catenin pathway. Table S1: the measurement of hair shaft elongation for Figures 3(a) and 4(d) (mm). Table S2: the sequences of the primers.
