# Correction to “Adipose‐Derived Stem Cell Exosomes Antagonize the Inhibitory Effect of Dihydrotestosterone on Hair Follicle Growth by Activating Wnt/*β*‐Catenin Pathway”

**DOI:** 10.1155/sci/9789426

**Published:** 2026-09-07

X. Tang, C. Cao, Y. Liang, et al., “Adipose‐Derived Stem Cell Exosomes Antagonize the Inhibitory Effect of Dihydrotestosterone on Hair Follicle Growth by Activating Wnt/*β*‐Catenin Pathway,” *Stem Cells International*, 2023, 5548112, https://doi.org/10.1155/2023/5548112.

In the article, there are errors in Figures 1g, 2d, 4b, and 6d.

In Figure 1g, the CD9 and CD81 bands appear identical, which the authors have confirmed was due to errors in figure preparation. The correct Figure 1 is shown below:

**Figure 1:**
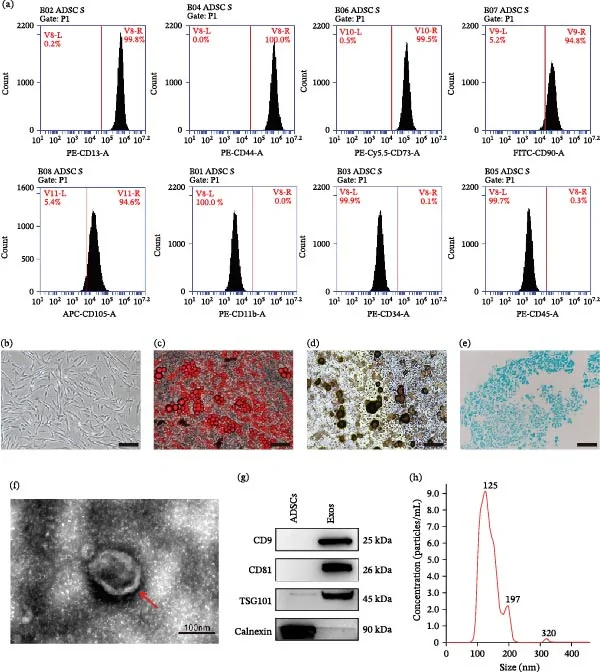
Identification of ADSCs and ADSC‐Exos. (a) Detection of surface markers of ADSCs by flow cytometry. (b) Cell morphology of human adipose stem cells, scale bar: 200 μm. (c) Lipogenic differentiation (Oil Red O staining), scale bar: 100 μm. (d) Osteogenic differentiation (Alizarin red staining), scale bar: 100 μm. (e) Chondrogenic differentiation (Alcian blue staining), scale bar: 100 μm. (f) Morphology of ADSC‐Exos observed by transmission electron microscopy, scale bar: 100 nm. (g) Detection of exosome surface markers by western blot. (h) ADSC‐Exos particle size distribution by NTA.

In Figure 2d, the significance level for the 20 µg/mL at 12 h should be  ^∗∗∗^ *p* < 0.001. The correct Figure 2 is shown below:

Figure 2ADSC‐Exos promotes the proliferation and migration of DPCs and enhances hair inducibility. (a) Uptake of PKH67 fluorescently labeled ADSC‐Exos by dermal papilla cells. Blue fluorescence: DAPI‐stained nuclei; green fluorescence: PKH67‐labeled ADSC‐Exos, scale bar: 100 μm. (b) CCK‐8 absorbance values of DPCs treated with different concentrations of ADSC‐Exos at 24, 48, and 72 h. (c) Scratch experimental plots of ADSC‐Exos treated DPCs, scale bar: 100 μm. (d) Quantification of scratch area in Figure 4b. (e) Graph of versican, ALP protein bands. (f) Relative quantitative graph of protein bands with grayscale values. (g) qPCR result of VEGF, HGF, BMP2, ALP, KGF, DKK‐1, and IGF‐1. The expression level of target genes was standardized to that of GAPDH. (h) Expression of HGF and IGF‐1 detected by ELISA in the culture supernatant of DPCs; *N* = 3,  ^∗^ *p*  <  0.05,  ^∗∗^ *p*  <  0.01,  ^∗∗∗^ *p*  <  0.001, and ^#^ *p* < 0.05, 10 μ*g*/mL compared with 20 μg/mL.

**Figure:**
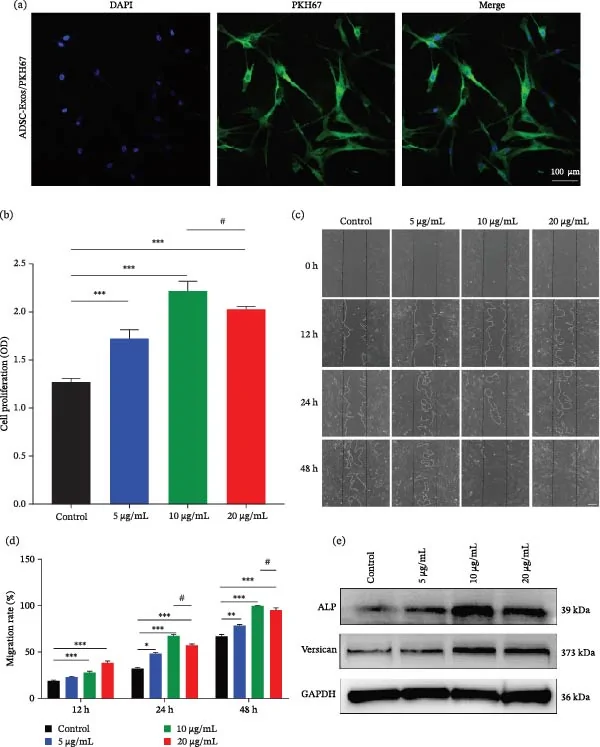

**Figure:**
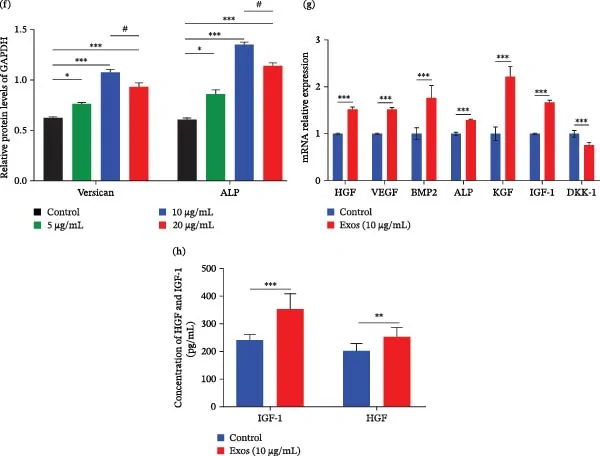

Moreover, in Figure 4b, there are unexpected, overlapping regions between the Exos and DHT + Exos panels at 0 h. The authors have confirmed that this was due to errors in figure preparation, and the DHT + Exos panel is incorrect. The correct Figure 4 is shown below:

Figure 4ADSC‐Exos antagonizes the inhibitory effect of DHT on hair follicle growth. (a) CCK‐8 absorbance values of ADSC‐Exos and/or DHT treated DPCs after 72 h. (b) Scratch experiment of ADSC‐Exos and/or DHT treated DPCs for 0, 12, 24, and 48 h, scale bar: 100 μm. (c) Quantitative analysis of scratch area in (d) hair follicle growth graph, *n* = 6. (e) Mean length of hair shaft increase (mm), *n* = 6. (f) Morphology of hair follicle growth at 0, 2, 4, 6, 8, and 10 days after ADSC‐Exos and/or DHT treatment, scale bar: 500 μm. (g) Ki67 expression of hair follicles in each group, scale bar: 100 μm. (h) The proportion of Ki67+ hair matrix keratinocytes in each group.  ^∗/#^ *p*  <  0.05,  ^∗∗/##^ *p*  <  0.01,  ^∗∗∗/###^ *p*  <  0.001. Comparison with  ^∗^control group and ^#^DHT group.

**Figure:**
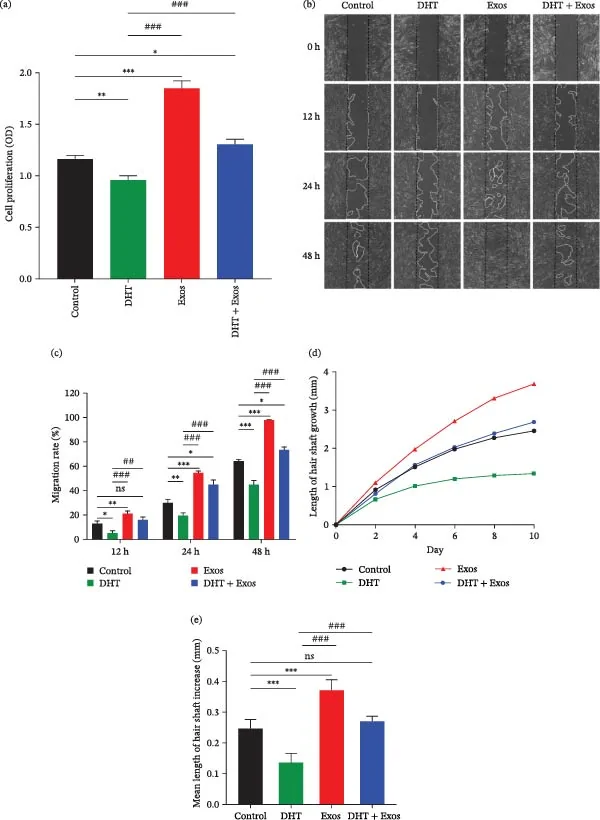

**Figure:**
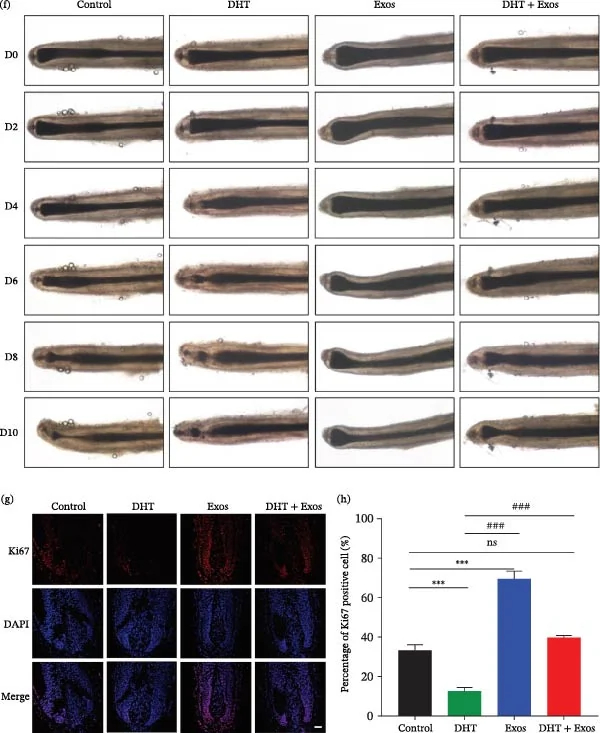

Lastly, errors were introduced during the production process in the 80 µg/mL column images of Figure 6d, in which incorrect white lines are present. The correct Figure 6 is shown below:

Figure 6ADSC‐Exos activates the Wnt/β‐catenin pathway by promoting pGSK‐3β (Ser9) and β‐catenin expression and β‐catenin nuclear translocation. (a) ADSC‐Exos upregulates the expression of pGSK‐3β and β‐catenin. (b) Relative quantification of protein bands in grayscale values. (c) Immunofluorescence staining of β‐catenin after treatment of DPCs with 0, 5, 10, 20 μg/mL of ADSC‐Exos, scale bar: 50 μm. (d) Expression levels of GSK‐3β, pGSK‐3β, and β‐catenin and nuclear transfer of β‐catenin in hair follicles of each group by immunofluorescence, scale bar: 100 μm. (e–g) Mean fluorescence intensity of total β‐catenin, nuclear β‐catenin, and pGSK‐3β/GSK‐3β in hair matrix region. (ns: *p* > 0.05,  ^∗^ *p*  <  0.05,  ^∗∗^ *p*  <  0.01, and  ^∗∗∗^ *p*  <  0.001).

**Figure:**
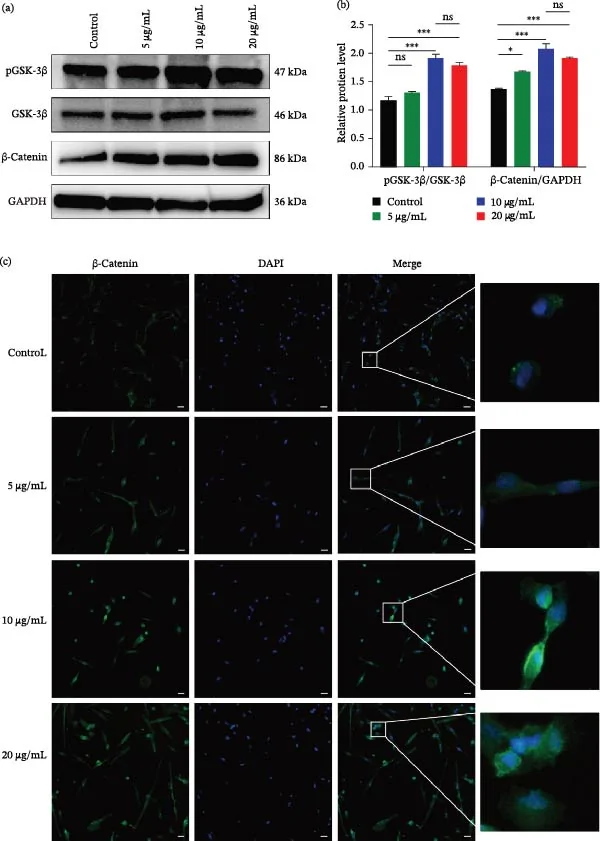

**Figure:**
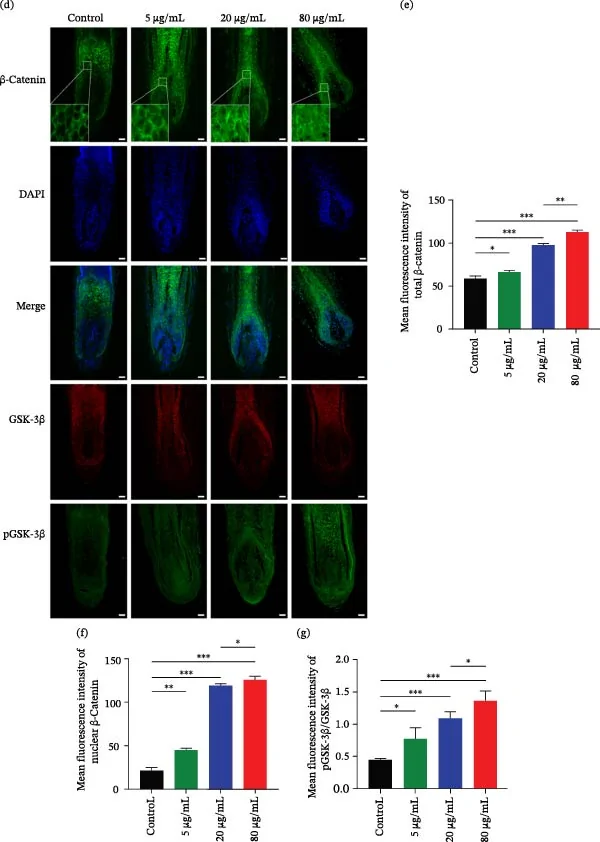

We apologize for these errors.

