# Supplementary material for: Adipose-Derived Stem Cell Exosomes Antagonize the Inhibitory Effect of Dihydrotestosterone on Hair Follicle Growth by Activating Wnt/β-Catenin Pathway
Source: Stem Cells Int. 2023 Sep 27;2023:5548112. doi: 10.1155/2023/5548112 (PMC10551537; doi:10.1155/2023/5548112)
Supplement: Supplementary Materials — Figure S1: retention of ADSC-Exos in C57BL/6 mice. Figure S2: qualitative morphological criteria to distinguish between anagen VI and catagen. In hair follicle organ culture anagen VI hair follicles show a hair matrix with a larger volume, a dermal papilla which is more onion-shaped and a melanin content which is maximal whereas catagen hair follicles have a thinner hair matrix, a dermal papilla which is often more oval and reduced melanin content. The melanin content of anagen hair follicles is higher than in catagen follicles. The percentage of Ki67+ cells of hair matrix keratinocytes in anagen VI hair follicles is significantly augmented. Figure S3: (a) the whole picture of hair shafts of Figures 3(c) and 4(f). Figure S4: HaCat cells were cultured with conditioned medium from DPCs treated with ADSC-Exos. Differentiation markers KRT40, MSX2, KRT5, and KRT15 were detected by qPCR; N = 3, ∗P < 0.05, ∗∗P < 0.01, and ∗∗∗P < 0.001. Figure S5: HE images with a full review of the injection sites of Figures 5(b) and 5(c). Figure S6: ADSC-Exos partially reverses the inhibitory effect of DHT on pGSK-3β. Figure S7: graphical abstract: ADSC-Exos were isolated from human adipose-derived stem cells by ultracentrifugation. ADSC-Exos promote the proliferation, migration and hair inducibility of DPCs, enhance the elongation of human hair follicles and accelerated telogen-to-anagen transition of C57/BL6 mice. ADSC-Exos counteracted the inhibitory effects of DHT on hair growth. Additionally, ADSC-Exos increased Ser9 pGSK-3β levels and facilitated nuclear translocation of β-catenin, which indicated that ADSC-Exos antagonize the inhibitory effect of DHT on hair follicle growth by activating Wnt/β-catenin pathway. Table S1: the measurement of hair shaft elongation for Figures 3(a) and 4(d) (mm). Table S2: the sequences of the primers. [file 5548112.f1.docx]

**Supplementary File**

**1. ADSCs identification.**

**(1) Surface markers**

Flow cytometry was used to observe the phenotypes of ADSCs. ADSCs were incubated with anti-CD11b-PE, anti-CD13-PE, anti-CD34-PE, anti-CD44-PE, anti-human-CD45-PE, anti-human-CD73-PE/Cy5.5, anti-CD90-FITC, anti-CD105-APC, anti-anti-HLA-DR-PE (Elabscience, China) at room temperature for 30min. Centrifuged at 1000 rpm for 5min to collect the cells, discard the supernatant, and washed the cells with pre-cooled PBS twice. Selected an appropriate channel, and the fluorescence of ADSCs was analyzed.

**(2) Multidirectional differentiation ability of ADSCs**

**Adipogenic differentiation:** ADSCs were seeded in 6-well plates (2×10^4^cells/cm^2^) and cultured in an incubator at 37°C and 5% CO2. Replaced medium every 3 days and changed Human Adipose-derived Stem Cell Adipogenic Differentiation Medium (Cyagen Biosciences, China) until cell fusion reached 100%. After 3 weeks, the medium for adipogenic induction differentiation was sucked up and rinsed with PBS twice. Added 2 mL 4% paraformaldehyde to each well and fix for 30 min. Oil Red O dye was added to stain for 30min, and results were detected with an inverted microscope.

**Osteogenic differentiation:** ADSCs were inoculated into 6-well plates coated with 0.1% gelatine (2×10^4^cells/cm^2^), and incubated at 37°C, 5% CO2. When the degree of cell fusion reached 60%-70%, the medium was replaced with Human Adipose-derived Stem Cell Osteogenic Differentiation Medium (Cyagen Biosciences, China) for osteogenic induction. After 2-4 weeks of induction, it was fixed with paraformaldehyde for 30min and dyed with Alizarin red for 3-5 min. The stained cells were observed under a microscope.

**Chondrogenic differentiation:** 3-4×10^5^ cells were transferred to a 15 mL centrifuge tube, centrifuged at 250 g for 4 min, then the supernatant was sucked out, and the cells were resuspended with Human Adipose-derived Stem Cell Chondrogenic Differentiation Medium (Cyagen Biosciences, China) for chondrogenic differentiation. After centrifuging at 150 g for 5 min, the tube cap was loose to facilitate gas exchange and incubated with 5% CO2 at 37 °C. The medium was changed every 2-3 days. After induction for 28 days, the cartilage spheres were fixed with paraformaldehyde, sectioned with paraffin embedding, and finally stained with Alcian blue solution. This Alcian blue stain showed the acid mucopolysaccharide in cartilage.

**2. Determination of ADSC-Exos treatment intervals in vivo.**

To detect the retention time of ADSC-Exos in mice and determine the interval of administration for subsequent in vivo experiments, we detected the retention of ADSC-Exos in C57BL/6 mice using DiR-labeled ADSC-Exos (ADSC-Exos/DiR) and in vivo fluorescence imaging. ADSC-Exos/DiR was injected into 4 subcutaneous parts of the depilation area after back depilation, and imaging was performed at 0.5, 24, 48 and 72h, respectively. As shown in Fig.7, a strong fluorescence signal was observed on the back of the mouse 24h after injection, which weakened after 48h and attenuated to less than 1/2 after 72h, indicating that ADSC-Exos remained in the back skin of the mouse for the longest 72h and was basically cleared or internalized by surrounding cells after 72h. Therefore, in order to maintain the appropriate therapeutic concentration of ADSC-Exos in vivo, the dosing interval is 48h.


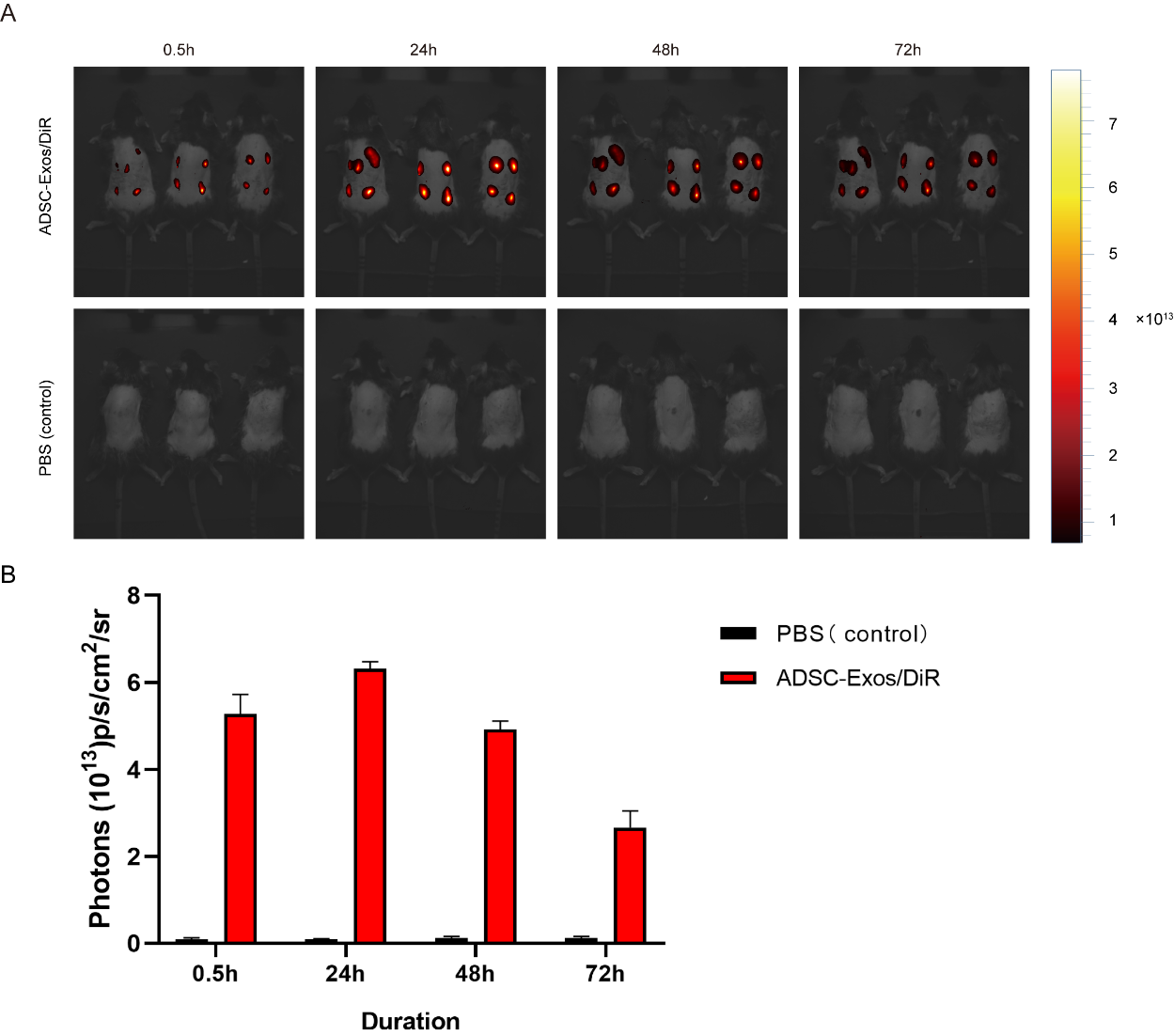


Supplementary Figure 1. Retention of ADSC-Exos in C57BL/6 mice. (A)Time-based in vivo fluorescent imaging of ADSC-Exos/DiR in C57BL/6 mice. ADSC-Exos/DiR or PBS (control) was administered intradermally after hair was clipped. (B) Quantification of fluorescent signals from mice, data are expressed as Means ± SD, radiant efficiency (photons/s/cm^2^/sr).

**3. Stage of the hair follicles in organ culture.**


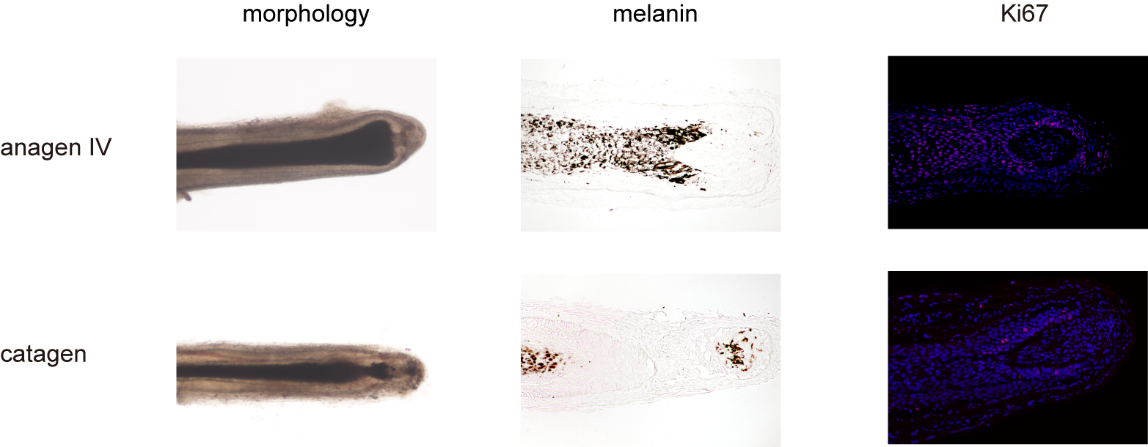


Supplementary Figure 2. Qualitative morphological criteria to distinguish between anagen VI and catagen. In hair follicle organ culture anagen VI hair follicles show a hair matrix with a larger volume, a dermal papilla which is more onion-shaped and a melanin content which is maximal whereas catagen hair follicles have a thinner hair matrix, a dermal papilla which is often more oval and reduced melanin content. The melanin content of anagen hair follicles is higher than in catagen follicles. The percentage of Ki-67 + cells of hair matrix keratinocytes in anagen VI hair follicles is significantly augmented.

**4. The whole picture of hair bulbs and shafts in organ culture.**


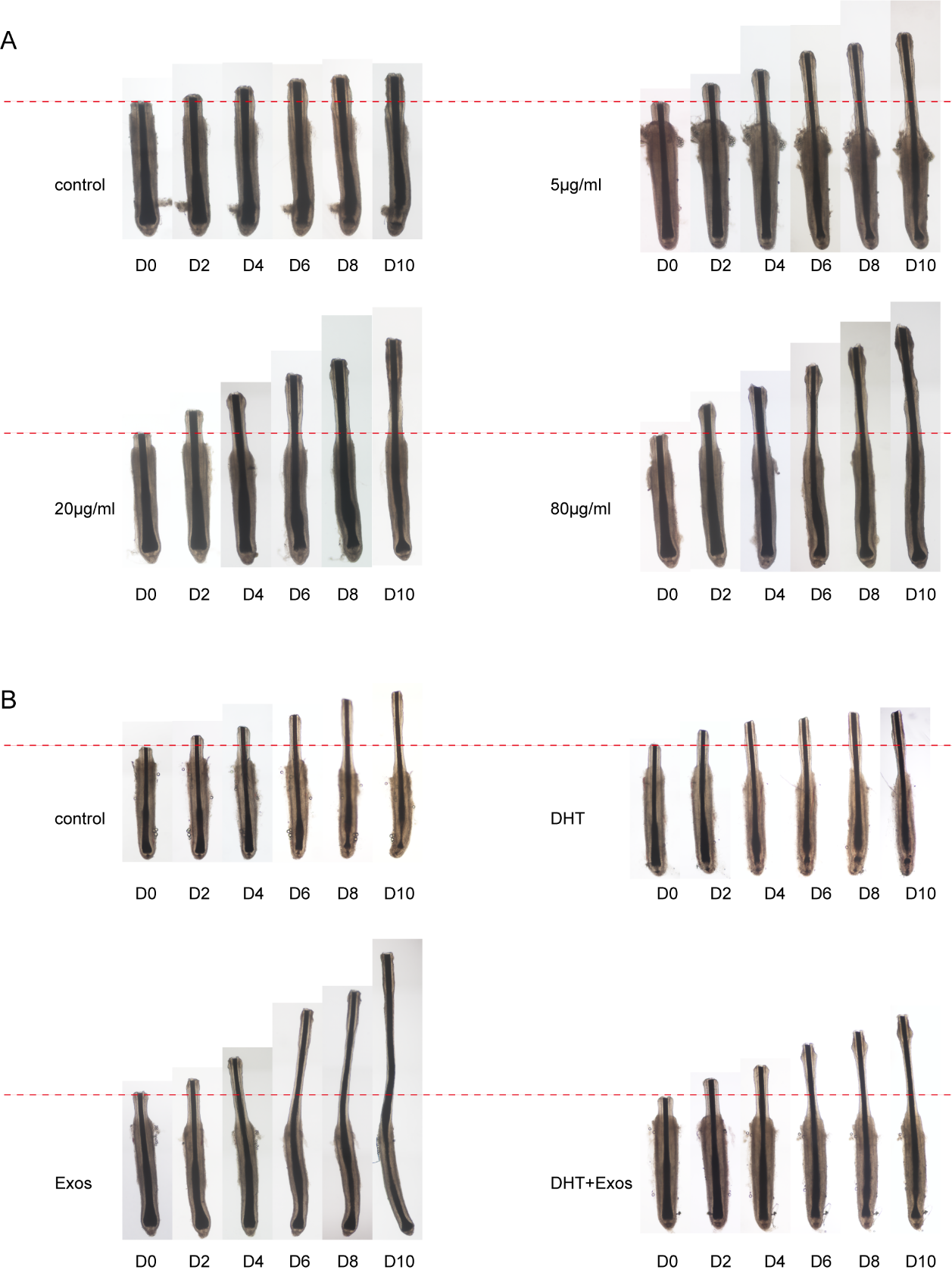


Supplementary Figure 3. (A)The whole picture of hair shafts of Figure 3C. (B)The whole picture of hair shafts of Figure 4F.

| Figure 3A |  |  |  |  |
| --- | --- | --- | --- | --- |
| Length of hair shaft growth (mm) | control | 5μg/ml | 20μg/ml | 80μg/ml |
| D0 | 0 | 0 | 0 | 0 |
| D2 | 1.87 | 2.04 | 2.16 | 2.2 |
| D4 | 3.04 | 3.32 | 3.59 | 3.76 |
| D6 | 4.07 | 4.5 | 4.83 | 5.02 |
| D8 | 4.79 | 5.28 | 5.79 | 6.055 |
| D10 | 4.98 | 5.89 | 6.53 | 6.86 |
|  |  |  |  |  |
|  |  |  |  |  |
| Figure 4D |  |  |  |  |
| Length of hair shaft growth (mm) | control | DHT | Exos | DHT+Exos |
| D0 | 0 | 0 | 0 | 0 |
| D2 | 1.83 | 1.34 | 2.21 | 1.635 |
| D4 | 3.04 | 2.03 | 3.955 | 3.1425 |
| D6 | 3.97 | 2.41 | 5.425 | 4.065 |
| D8 | 4.55 | 2.59 | 6.625 | 4.795 |
| D10 | 4.92 | 2.69 | 7.375 | 5.3875 |

Supplementary Table 1. The measurement of hair shaft elongation for Figure 3A and Figure 4D (mm).

**5. Functional validation of DPCs on keratinocyte differentiation.**


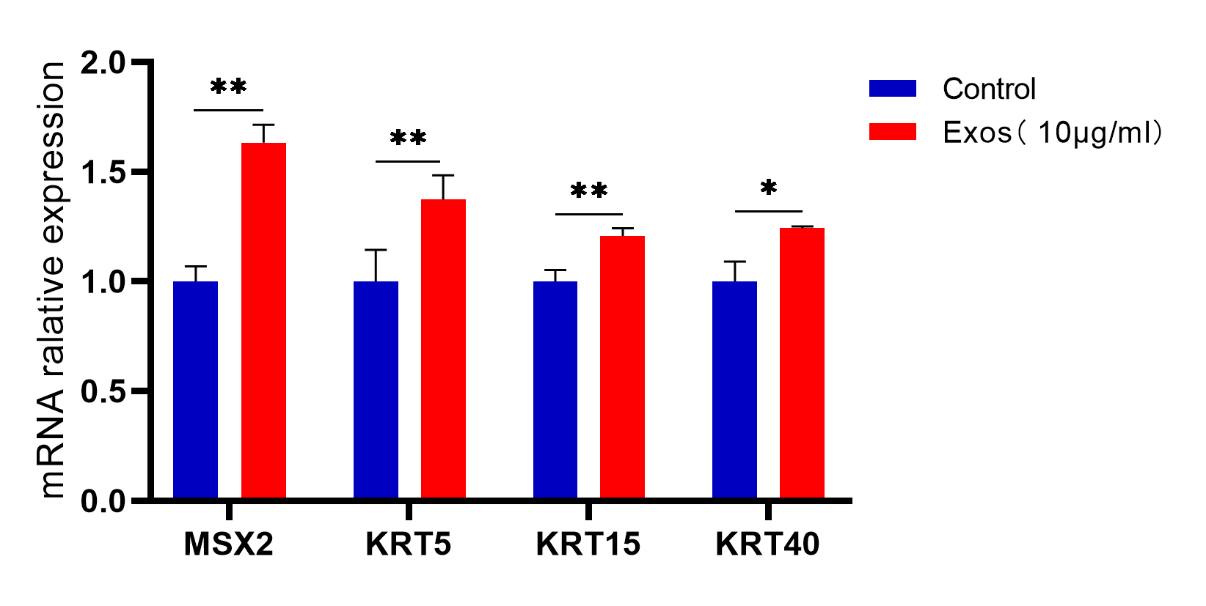


Supplementary Figure 4. HaCat cells were cultured with conditioned medium from DPCs treated with ADSC-Exos. Differentiation markers KRT40, MSX2, KRT5, and KRT15 were detected by qPCR. N = 3. *:P<0.05, **:P<0.01, ***:P<0.001.

**6. HE images with full review of the mice skin.**


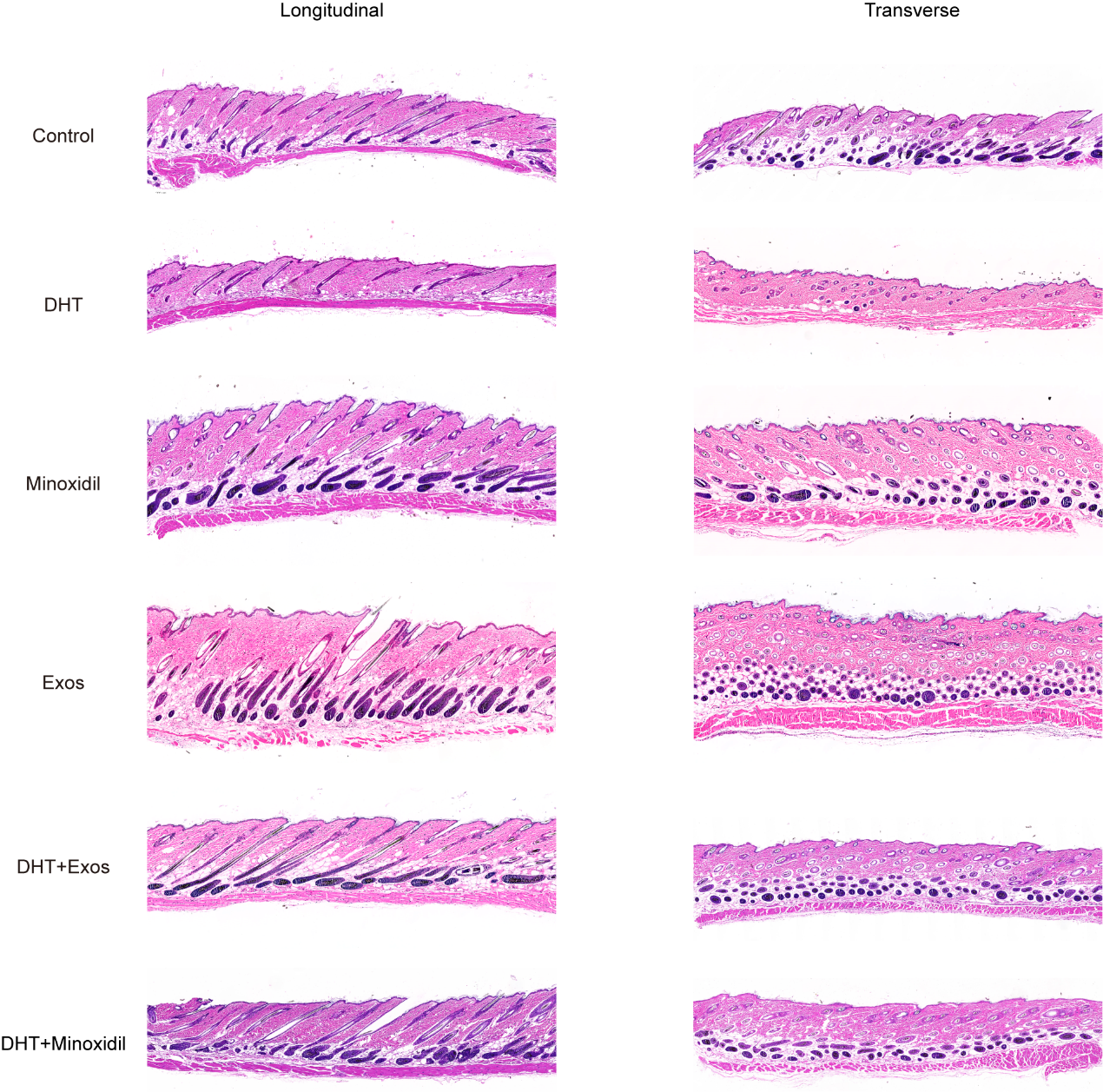


Supplementary Figure 5. HE images with a full review of the injection sites of Figure 5B and 5C.

**7. The sequences of the primers.**

| Gene | Forward | Reverse |
| --- | --- | --- |
| IGF-1 | 5′-GCTCTTCAGTTCGTGTGTGGA-3′ | 5′ -GCCTCCTTAGATCACAGCTCC-3′ |
| HGF | 5′-GCTATCGGGGTAAAGACCTACA-3′ | 5′-CGTAGCGTACCTCTGGATTGC-3′ |
| VEGF | 5′-GTCCAACTTCTGGGCTGTCT-3′ | 5′-CCCTCTCCTCTTCCTTCTCTTC-3′ |
| BMP-2 | 5′-ACTACCAGAAACGAGTGGGAA-3′ | 5′-GCATCTGTTCTCGGAAAACCT-3′ |
| KGF | 5′-TCCTGCCAACTTTGCTCTACA-3′ | 5′-CAGGGCTGGAACAGTTCACAT-3′ |
| DKK-1 | 5′-CAATGGTCTGGTACTTATTCCCG-3′ | 5′-CAATGGTCTGGTACTTATTCCCG-3′ |
| ALP | 5′-ACTGGGGCCTGAGATACCC-3′ | 5′-TCGTGTTGCACTGGTTAAAGC-3′ |
| KRT-5 | 5′ -AGGAGTTGGACCAGTCAACAT-3′ | 5′ -TGGAGTAGTAGCTTCCACTGC-3′ |
| KRT-15 | 5′ -GACGGAGATCACAGACCTGAG-3′ | 5′ -CTCCAGCCGTGTCTTTATGTC-3′ |
| KRT-40 | 5′ -TCCGTGGAAACAGCTTGTCTC-3′ | 5′ -GCAGGGACTATTACAACTCCCA-3′ |
| MSX-2 | 5′ -ATGGCTTCTCCGTCCAAAGG-3′ | 5′ -CGGCTTCTTGTCGGACATGA-3′ |
| GAPDH | 5′ -AGGGCTGCTTTTAACTCTGGT-3′ | 5′ -CCCCACTTGATTTTGGAGGGA-3′ |

Supplementary Table 2. The sequences of the primers.

**8. ADSC-Exos partially reverses the inhibitory effect of DHT on pGSK-3β.**


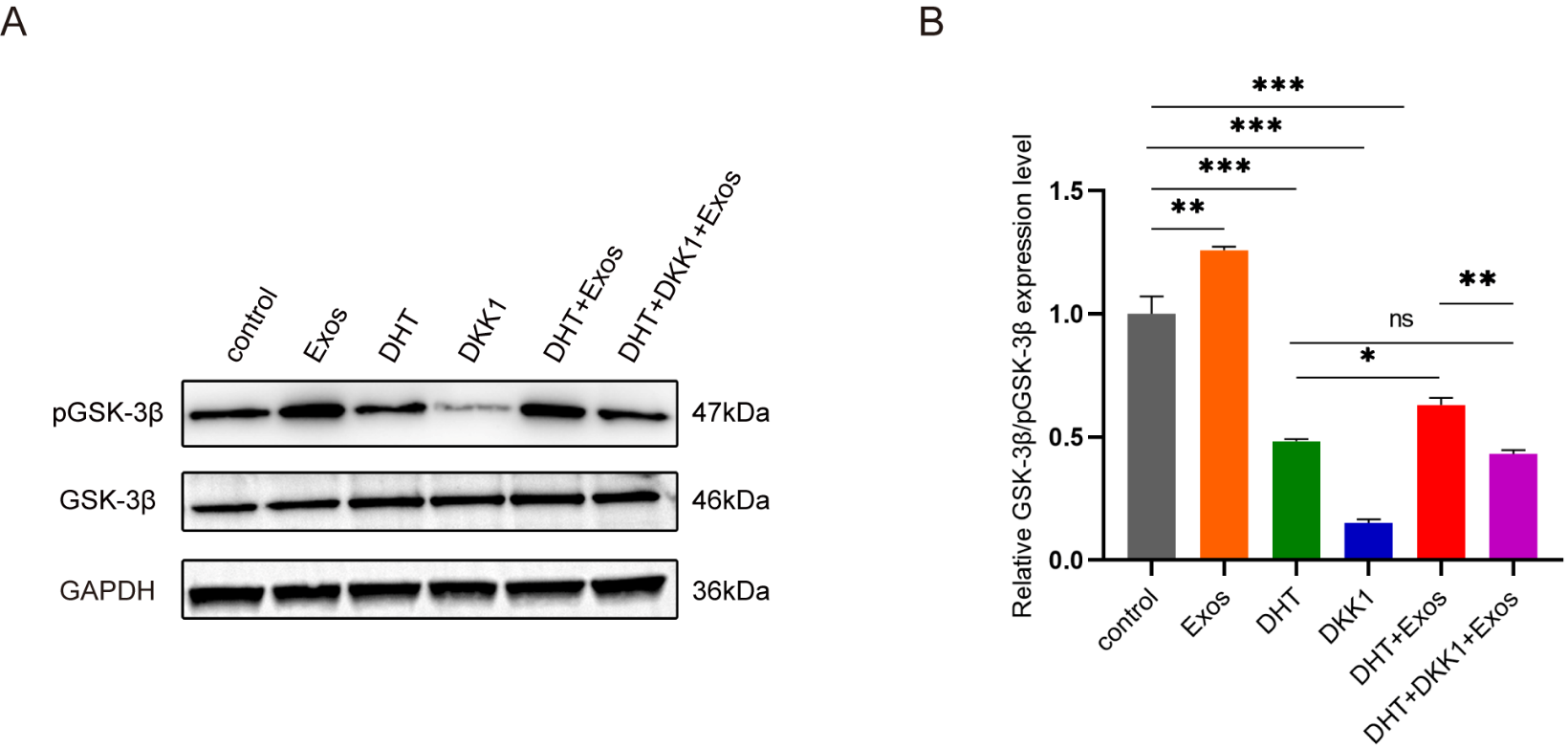


Supplementary Figure 6. ADSC-Exos partially reverses the inhibitory effect of DHT on pGSK-3β.

**9.** **Graphical Abstract**


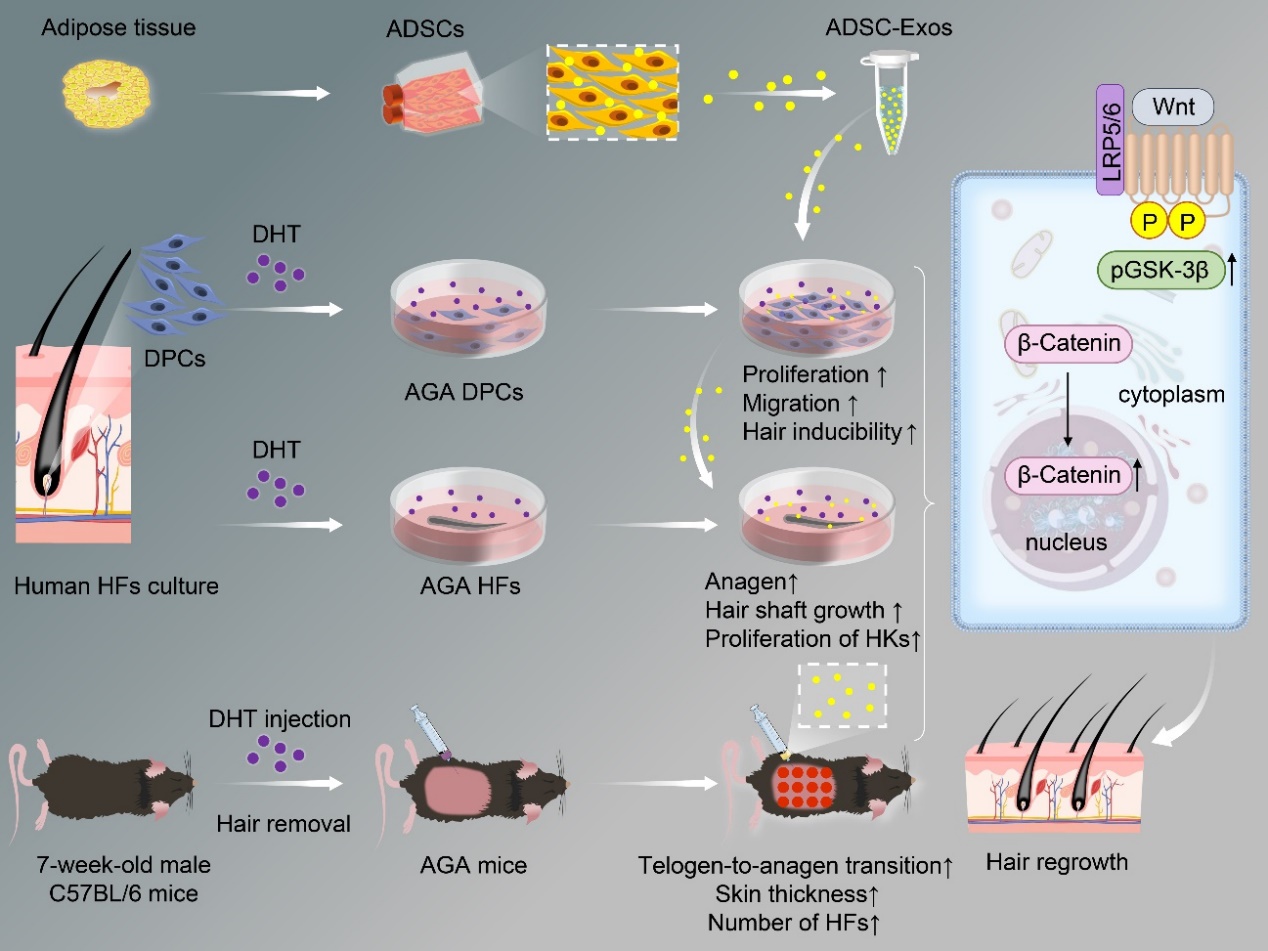


Supplementary Figure 7. ADSC-Exos were isolated from human adipose-derived stem cells by ultracentrifugation. ADSC-Exos promoted the proliferation, migration and hair inducibility of DPCs, enhanced the elongation of human hair follicles and accelerated telogen-to-anagen transition of C57/BL6 mice. ADSC-Exos counteracted the inhibitory effects of DHT on hair growth. Additionally, ADSC-Exos increased Ser9 pGSK-3β levels and facilitated nuclear translocation of β-catenin, which indicated that ADSC-Exos antagonized the inhibitory effect of DHT on hair follicle growth by activating Wnt/β-catenin pathway.
